# Simulation of herbicide impacts on a plant community: comparing model predictions of the plant community model IBC-grass to empirical data

**DOI:** 10.1186/s12302-018-0174-9

**Published:** 2018-11-14

**Authors:** Jette Reeg, Simon Heine, Christine Mihan, Sean McGee, Thomas G. Preuss, Florian Jeltsch

**Affiliations:** 1 Department of Plant Ecology and Nature Conservation, University of Potsdam, Am Mühlenberg 3, 14476 Potsdam, Germany; 2 Bayer AG, Alfred-Nobel-Str. 50, 40789 Monheim am Rhein, Germany; 3 Bayer CropScience LP, Research Triangle Park, NC 27709 USA; 4 Berlin-Brandenburg Institute of Advanced Biodiversity Research (BBIB), 14195 Berlin, Germany

**Keywords:** Plant community model, Non-target terrestrial plants, Community-level effects, Herbicide risk assessment, Individual-based modeling

## Abstract

**Background:**

Semi-natural plant communities such as field boundaries play an important ecological role in agricultural landscapes, e.g., provision of refuge for plant and other species, food web support or habitat connectivity. To prevent undesired effects of herbicide applications on these communities and their structure, the registration and application are regulated by risk assessment schemes in many industrialized countries. Standardized individual-level greenhouse experiments are conducted on a selection of crop and wild plant species to characterize the effects of herbicide loads potentially reaching off-field areas on non-target plants. Uncertainties regarding the protectiveness of such approaches to risk assessment might be addressed by assessment factors that are often under discussion. As an alternative approach, plant community models can be used to predict potential effects on plant communities of interest based on extrapolation of the individual-level effects measured in the standardized greenhouse experiments. In this study, we analyzed the reliability and adequacy of the plant community model IBC-grass (individual-based plant community model for grasslands) by comparing model predictions with empirically measured effects at the plant community level.

**Results:**

We showed that the effects predicted by the model IBC-grass were in accordance with the empirical data. Based on the species-specific dose responses (calculated from empirical effects in monocultures measured 4 weeks after application), the model was able to realistically predict short-term herbicide impacts on communities when compared to empirical data.

**Conclusion:**

The results presented in this study demonstrate an approach how the current standard greenhouse experiments—measuring herbicide impacts on individual-level—can be coupled with the model IBC-grass to estimate effects on plant community level. In this way, it can be used as a tool in ecological risk assessment.

**Electronic supplementary material:**

The online version of this article (10.1186/s12302-018-0174-9) contains supplementary material, which is available to authorized users.

## Background

With agricultural landscape covering almost half of the European land area, it is reasonable that environmental impact of agricultural practices is evaluated. Of particular interest are the potential impacts of pesticide applications, which regulatory authorities around the world are required to evaluate and make regulatory decisions on the acceptability of potential risks to the environment. Pesticides are designed to control pests, including competing weed species in agricultural fields, thereby increasing the yields. However, small amounts of these pesticides may reach adjacent off-field areas, the so-called non-target areas [1, 2]. To prevent undesired effects of an herbicide application, the registration and application are regulated by risk assessment schemes in many industrialized countries [3]. To characterize the effects of herbicide loads potentially reaching off-field areas on plants, standardized individual-level greenhouse experiments are conducted on a selection of crop and wild plant species [4–7].

To account for uncertainties associated with extrapolating from testing plant individuals in the greenhouse to plant communities in the field, an assessment factor may be applied. However, the appropriateness of the assessment factor can be debated as currently there is no reference tier that would allow for calibration. Extending the standard ecotoxicological tests for non-target terrestrial plants under worst-case greenhouse conditions to more realistic field conditions or community level is not feasible. Not only reproducibility is a major constraint, but there is also the question of representativeness of higher tier studies for different landscapes under different climatic conditions. Additional data are needed to reduce uncertainty associated with predicting the potential long-term impacts on non-target terrestrial plant communities from short-term individual-level greenhouse studies.

Several researchers investigated specific uncertainties associated with the current standard test guidelines [4, 5]. Many of these focus on comparing individual-level standard experiments conducted in greenhouses versus in the field. They assess the level of protection when using greenhouse experiments to predict expected effects under field conditions [8–10]. Although it was generally shown that the effects on single species observed in the greenhouse are more pronounced than under field conditions and, therefore, lead to a conservative risk assessment, these experiments cannot illustrate the influence of competition between individuals of different plant species. Only few studies focused on artificial communities to account for these processes [11, 12]. Both studies are based on a small species pool (4 and 6 plant species) and thus do not represent the diversity and composition of plant communities observed in environments that may receive off-site herbicide exposure. Real field studies testing the impact of in-field herbicide application on plant communities adjacent to the arable field are rare (e.g., [13, 14]). Thus, general conclusions of the herbicide impact on plant communities under various environmental conditions cannot be made.

In nature conservation, modeling approaches are frequently used to overcome the limits of experimental studies and make general predictions on long-term impacts of, for example, climate change or grazing intensity [15]. Cousins et al. [16] highlight that landscape models are a useful method to increase the understanding of mechanisms affecting grassland communities due to land use change. Such ecological models have the advantage to cover a variety of different environmental scenarios and therewith a wider range of potential impacts than empirical studies, which are often limited in space and time. These studies highlight that modeling approaches can be valuable tools to address uncertainties in the current risk assessment scheme by analyzing potential long-term impacts on community level.

In this study, we analyzed the reliability and adequacy of the plant community model IBC-grass by comparing model predicted and experimentally measured effects at the plant community level. IBC-grass is an individual-based and spatially explicit plant community model; thus, individual-level effects from standard greenhouse studies can be integrated and competition between plant individuals is accounted for. We adjusted the model to the settings in the empirical study of Reuter and Siemoneit-Gast [12]; using one part of the data set for calibration (control data and dose–responses after herbicide application of the monocultures on individual plant basis). We evaluated if the calibrated model is able to predict similar plant community-level effects as observed in the empirical data (second part of the data set) and analyzed to which degree the model is able to reproduce realistic effects by calculating model adequacy and reliability as a measure for the model fit [17].

## Methods

### Short summary of the experimental study design

Based on the results of a pre-study in which the researchers tested the germination rate and handling of plants, Reuter and Siemoneit-Gast [12] tested their proposed higher tier study design on 6 wild plant species: *Bromus erectus*, *Cynosurus cristatus*, *Galium mollugo*, *Leontodon hispidus*, *Silene nutans* and *Trifolium pratense*. Plant individuals were transplanted into monocultures and communities after reaching the growth stage BBCH 12–14. In the monoculture setup, 4 individuals of the same plant species were transplanted into a pot with a diameter of 7 cm. In the artificial communities, 8 individuals per plant species were transplanted randomly in square pots of 17 × 17 cm. The distance between each individual was 2.5 × 2.6 cm. The remaining space in the center of each pot was planted with an individual of a randomly chosen species. Monoculture setup included 4 repetitions for each of the 3 assessment dates (i.e., overall 12 pots per treatment); community setup included 3 repetitions per assessment date (i.e., overall 9 pots per treatment). The researchers used the experimental setup to investigate two different herbicides: a broad spectrum herbicide, RoundUp^®^ (active ingredient glyphosate), and a selective herbicide, Monitor^®^ (active ingredient sulfosulfuron). Five different test item rates (3, 5, 9, 15, 25% of the maximal application rate of 3 L/ha RoundUp^®^ and 5, 9, 17, 31 and 55% of the maximal application rate of 25 g/ha of Monitor^®^) and a control were tested per herbicide. In the EU, the current off-field risk assessment approach assumes 2.77% of an application in field crops might drift of the target application site. This drift rate accounts for normal farming practice and machine operation and assumes wind direction into the off-field area. The lowest rates tested were in the same range (RoundUp^®^) or higher (Monitor^®^) than the rates that would be used in a baseline EU risk assessment to assess the potential risk from exposure to off-field areas in 1 m distance. Fresh shoot weight and phytotoxicity were measured every 2 weeks over 6 weeks; however, only results for shoot weight could be compared to the model predictions, as the model is designed and developed to simulate biomass and not phytotoxicity. In addition, the assessment of phytotoxicity is a very subjective measure and a conversion of symptoms into effects on biomass would not be feasible. For more details, see Reuter and Siemoneit-Gast [12].

### IBC-grass

The spatially explicit and individual-based plant community model IBC-grass was originally designed to test the response of plant communities to different disturbances such as grazing [18–22]. The main processes such as inter- and intraspecific competition for space and resources, growth, mortality and disturbances like grazing, trampling, mowing and herbicide impact are accounted for. A detailed description of the IBC-grass version on which this study is based on can be found in the appendix of Reeg et al. [21]. It follows the ODD (overview, design concept and details) protocol [23]. Here, we will give only a short overview of the main aspects and focus on the modifications and adaption we integrated in the model to reconstruct the exposure scenario from the study of Reuter and Siemoneit-Gast [12] to evaluate the precision of the model predictions.

#### General description of the main principles and processes

##### Plant functional type approach

To allow for general conclusions, plant species are classified into plant functional types (PFTs) according to important trait characteristics. This functional type approach is widely used in community ecology to explain dynamics in ecosystems [24]. Experimental studies proved that the response of plant species with similar trait characteristics to environmental conditions and disturbances is comparable. Six different traits and trait syndromes (i.e., a group of traits representing general trade-offs) are distinguished in IBC-grass: growth form, plant size (correlated with seed mass, and dispersal traits), resource response (correlation of competitive ability and stress-tolerance), grazing response and clonality. Plant species of the species pool of interest are classified into PFTs based on trait information in the databases BiolFlor, LEDA and cloPla3 [25–27].

##### Zone of influence approach

Intra- and interspecific competition is accounted for in the aboveground and in the belowground compartment. Plant individuals acquire resources within a circular area around the stem—their zone of influence (ZOI). For the belowground compartment, the size of the ZOI is determined only by the root biomass. It is assumed that plants have similar root geometries. Aboveground, the ZOI of a plant is determined by the shoot biomass and shoot geometry accounting for taller plants and shading effects. In overlapping ZOI areas, plant individuals compete for resources. Belowground, competition is simulated size-symmetrically. Thus, the distribution of resources in overlapping areas only depends on the competitive ability of the PFTs (resource response traits). Aboveground, resource competition is partially size-asymmetrically accounting for shading effects of taller plants. For both compartments, intraspecific competition is stronger than interspecific competition.

##### General processes

**Fig. 1:**
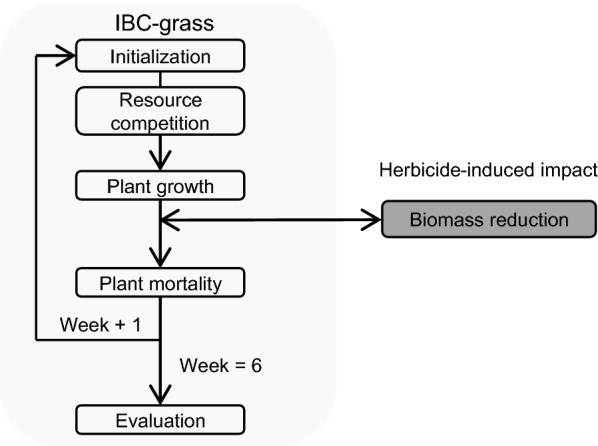
Flowchart of the processes in the current version of IBC-grass. Several processes such as seed production, seed dispersal and biotic disturbances such as grazing are omitted in this version due to the short time scale of the experiments

Figure 1 gives a general overview of all processes accounted for in this current IBC-grass version. Several processes important for long-term community dynamics are excluded in this version due to the short-term time scale of 6 weeks in the experimental study. In the following, the main processes applied in this version of IBC-grass are explained. For more details, see the appendix of Reeg et al. [21].

*Plant growth and mortality* As mentioned before, plants acquire resources within their ZOI and compete for resources in overlapping areas. The acquired resources are allocated to the roots and shoot, and converted into biomass based on a constant conversion rate, the current shoot (root) mass, the trait characteristics of the shoot (root), the growth form, the maximal resource utilization and the maximal plant mass. In the absence of competition, the growth function results in a sigmoid growth (see also in [28, 29]). Since the experimental study traced plant biomass over a time frame of 6 weeks and the researchers did not observe any mortality, we excluded mortality in this version. However, it is an important process for long-term community dynamic. A detailed description of how mortality is modeled in IBC-grass can be found in the ODD protocol of Reeg et al. [21].

*Seed dispersal, germination and establishment* These processes are important for community dynamics. However, since the time frame of the experimental study is limited to 6 weeks, these processes have no impact on the results. The young plant individuals are not yet producing seeds. In this specific study, we used a 100% germination and establishment rate for the plant individuals (see Spatial set up in model calibration to experimental data). A detailed description of the implemented process can be found in the ODD protocol in the appendix of Reeg et al. [21].

*Biotic disturbances* In this version, we excluded all biotic disturbances, such as grazing, tramping and cutting. We only integrated the herbicide impact.

*Abiotic factors and temporal dimensions* IBC-grass distinguishes aboveground and belowground resource availability. For both compartments, resources are distributed homogenously in space and time. One time step in the model represents 1 week.

### Model preparation

#### Spatial setup

We adjusted the spatial initial setup in the model to mimic the experimental setup (Table 1). The grid size of the model was set to 7 × 7 grid cells, representing a 7 × 7 cm^2^ pot, for the monocultures, and 20 × 20 grid cells, representing a 20 × 20 cm^2^ pot for the artificial communities. In the monoculture setup, we distributed 4 seeds of the same PFT on the grid. Seeds had a distance of 2 grid cells to one another. The 4 seeds germinated with a probability of 100%, resulting in 4 young plant individuals located in the grid. In the model, herbicide treatment started according to the time of transplanting in the experiments (Table 2). The spatial setup in the modeled communities was adjusted according to that in the experiments: for each species, 6 plant individuals were initialized randomly in the grid with a distance of 2 grid cells to each other. The grid cell located in the middle was initialized with a randomly chosen plant individual. As mentioned before, initial plant biomasses in the communities were based on the biomasses in the monoculture set up (in the model) at the time of reaching the BBCH 12–14 in the experiment (Table 2).

**Table 1:** Overview of the experimental setup compared to the model set up

	IBC-grass	Experimental study
Monoculture		
Plot size	7 × 7 cm^2^	Ø 7 cm
Nb. of individuals	4	4
Distance between individuals	2 cm (2 grid cells)	No information
Community		
Plot size	20 × 20 cm^2^	17 × 17 cm^2^
Nb. of individuals	49	49
Distance between individuals	2 cm (2 grid cells)	2.5 × 2.6 cm

Since IBC-grass simulates only rectangular plots, it is not able to represent a circular pot of 7 cm diameters. As the model assumes that plant individuals cannot grow beyond the plot size, we chose to use a greater area (7 × 7 cm) rather than a smaller one (6 × 6 cm) to account for potential shoot growth beyond the pot size. There was no information about the distance between plant individuals within the monoculture setup of the experiment. Therefore, we decided to use a similar distance as in the community set up. This also results in an even distribution of individuals within the plot

**Table 2:** Compared biomasses in the model and the experiment

Species	Week > 75% emergence	Week BBCH 12–14	Age at potting in the experiment (‘week 0’ in the modeled monocultures)
*B. erectus*	2	4	2
*C. cristatus* ^a^	2	2	0
*G. mollugo*	2	3	1
*L. hispidus*	2	3	1
*S. nutans*	3	4	1
*T. pratense*	1	3	2

Due to the different germination times and growth rates, the age of the plant individuals when being transplanted in the experiments differs between species. e.g., *B. erectus* was transplanted at the age of 2 weeks. In this case, herbicide treatment in the simulated monoculture started 2 weeks after germination and initial biomasses in the community setup are based on these biomasses (biomasses 2 weeks after germination in the simulated monocultures). In the analyses of *B. erectus* in the monoculture, the simulated biomasses of plants 4, 6 and 8 weeks after germination are equivalent to the biomass measurements of week 2, 4 and 6 in the experiments

^a^BBCH12-14 is reached between 2 and 3 weeks

#### Model calibration

In the following section, we will describe the process of calibrating the model against the monoculture control and effect data. All model parameters and settings are summarized in Additional file 1: Appendix A.

##### Resource levels in IBC-grass

As the model is not considering concrete resources such as nutrients, soil moisture or light, but groups all kinds of resources into overall resource units, we cannot specify resources in a numerical way, e.g., % CaCO3. To find the resource unit levels for the aboveground and belowground compartment, which result in similar growth patterns as in the experiments, we used Latin Hypercube Sampling (LHS, [30]). Therewith, we uniformly covered the whole potential sampling space. We selected these resource levels, for which the simulated shoot masses fell within the empirically measured shoot masses. We used only the monoculture control data for this calibration step, in order to have an independent validation on the community growth. Additionally, although Reuter and Siemoneit-Gast [12] used different soils for the two herbicides and conducted the studies in spring and in summer, we combined the shoot masses for all control monocultures to have a higher sampling size (*N* = 8). This process is based on the pattern-oriented modeling approach [31, 32], which aims at comparing the patterns predicted by a model with patterns observed in the nature, e.g., the temporal growth of plants.

We ended up varying belowground resource units between 60 and 120 (medium and high resource level) and aboveground resource units between 50 and 100 (medium and high resource level). 90 different resource combinations were selected using Latin Hypercube Sampling (LHS, [30]), therefore, covering uniformly the sampling space. Each resource combination was repeated ten times to account for stochasticity, resulting in 900 simulation runs.

##### Species classification into PFTs

We classified the six plant species according to the categories used in Weiss et al. [19] and Reeg et al. [21] (Table 3). Without any further adaption, the monoculture control biomass of *L. hispidus*, *T. pratense* and *S. nutans* could not be sufficiently predicted by the model. The experimental biomass of *T. pratense* was even higher than the maximal plant masses assumed in the model according to the classification. Also for *L. hispidus* the modeled biomass was not able to increase further due to the maximal plant size assumed in the trait characterization. Thus, we increased the maximal plant mass of these two species to the next higher category (from small to medium sized plant species). This can be also supported by data found in the TRY database for maximal plant size [33]. According to the root/shoot ratios found in the trait database [34–36] and according to expert knowledge (Michael Ristow, University of Potsdam, Germany, personal communication, 2017), *S. nutans* allocates more resources to root growth compared to other plant species, especially in early live stages (approx. 50% higher root biomass than shoot biomass [34]). In contrast to that, *T. pratense* has higher shoot biomasses (approx. 20%, [35, 36]). Based on this knowledge, we integrated a root and shoot allocation factor in the model (Eqs. 1a, b; Eqs. 2a, b).

**Table 3:** Classification of species into plant functional types (PFT) according to the classification rules [21] and current adaptations

Species	Plant size	Growth form	Resource response	Grazing response	Root/shoot allocation
*B. erectus*	Large	Semi-rosette	Stress-tolerator	Tolerator	Alloc_root/shoot = 1
*C. cristatus*	Large	Semi-rosette	Intermediate	Avoider	Alloc_root/shoot = 1
*G. mollugo*	Medium	Erect	Competitor	Intermediate	Alloc_root/shoot = 1
*L. hispidus*	Medium^a^	Rosette	Intermediate	Tolerator	Alloc_root/shoot = 1
*S. nutans*	Medium	Semi-rosette	Intermediate	Intermediate	Alloc_shoot = 1 Alloc_root = 0.5
*T. pratense*	Medium^a^	Semi-rosette	Competitor	Tolerator	Alloc_shoot = 0.2 Alloc_root = 1

During the calibration process (i.e., fitting simulated shoot growth against empirical shoot growth in monoculture controls) a root/shoot allocation trait was integrated in the model. The trait characteristics are based on trait data (‘root/shoot ratio’) and expert knowledge

^a^According to classification rules the species would be classified as small. However, experimental data reach or exceed the maximal plant size even within 6 weeks of growth. Therefore, we classified these species in the next higher category

Higher resource allocation into the shoot growth was simulated as:

1a $${\text{Shoot}}\_{\text{resources }} = {\text{ Shoot}}\_{\text{resources }} + \, \left( {{\text{Root}}\_{\text{resources }}*{\text{alloc}}\_{\text{shoot}}} \right)$$

1b $${\text{Root}}\_{\text{resources }} = {\text{ Root}}\_{\text{resources}}*\left( { 1- {\text{alloc}}\_{\text{shoot}}} \right)$$

Higher resource allocation into the root growth was simulated as:

2a $${\text{Shoot}}\_{\text{resources }} = {\text{ Shoot}}\_{\text{resources }}*\left( { 1- {\text{alloc}}\_{\text{root}}} \right)$$

2b $${\text{Root}}\_{\text{resources }} = {\text{ Root}}\_{\text{resources }} + \, \left( {{\text{Shoot}}\_{\text{resources }}*{\text{alloc}}\_{\text{root}}} \right)$$

With *Shoot/Root_resources*: the resources allocated to root and shoot growth without the allocation factor and *alloc_shoot/root*: the PFT-specific allocation factor.

These factors allow for an additional shift in resource allocation from shoots to roots and vice versa after the general distribution of resources between roots and shoots. For *T. pratense*, 20% of the resources assigned for root growth were additionally available and shifted to shoot growth. In the case of *S. nutans*, 50% of the resources assigned for shoot growth were additionally available and shifted to root growth.

##### Integration of herbicidal effects

The design of the empirical study was based on the OECD Guideline for Vegetative Vigor studies [5] and focused on the endpoint biomass, not measuring the effect on seedling emergence or survival. This means, plant individuals were oversprayed with different application rates and fresh weight was measured 2, 4 and 6 weeks after application. Therefore, we integrated the herbicide effect in the model only as a reduction in biomass gain per weekly time step.

The herbicide effect was based on the effects on fresh weight (reduction in shoot mass) measured 4 weeks after application in the experimental monocultures. We selected the 4th week to be as close as possible to the standardized greenhouse experiments, which measure effects on biomass 3–4 weeks after herbicide application. For each species and herbicide, the dose–response curves were calculated using an optimization algorithm [37], which calculates the ER50 and slope (b) estimates of the dose–response function (Eq. 3):

3 $${\text{Effect}} \;\left( {{\text{Application }}\;{\text{rate}}} \right) = \frac{{{\text{Application}} \;{\text{rate}}^{b} }}{{{\text{ER50}}^{b} + {\text{Application}}\; {\text{rate}}^{b} }}$$

Effect is the reduction in growth for the specific application rate, application rate is the applied rate of the herbicide [in g/ha for Monitor^®^ or mL/ha for RoundUp^®^], *ER50* is the rate [in mL/ha for Monitor^®^ or g/ha for RoundUp^®^], at which 50% reduction of biomass occurred, and *b* is the slope for the dose–response function. Effect and slope *b* are dimensionless.

In each weekly time step following the simulated herbicide application in the model, the biomass gain was reduced by this species and dose specific effect based on the dose–response data. We assumed no dissipation of the herbicides throughout the time of the experiment, i.e., the effect does not change over time. This holds true for both the modeled monocultures and communities: Modeled effects are based on these species-specific dose responses and applied each week after herbicide application.

#### Analyses

For model calibration and the comparison of the aboveground biomasses without herbicide effect, we used pattern-oriented modeling—the visual comparison of the patterns (i.e., shoot mass dynamics over time) observed in the empirical data compared to those in the modeled simulations. Therefore, we first needed to convert the empirically measured fresh weights to dry weight, which is the biomass parameter simulated in IBC-grass.

We repeated the control monoculture experiment to measure the fresh to dry weight ratio for each species and used the mean ratio as a conversion factor (see Additional file 1: Appendix B for further details). Besides pattern-oriented calibration, we calculated the Welch Two Sample *t* test (not paired, no correction for multiple comparison, alpha value = 0.05) for each experimental and modeled pair (by PFT and time) of data to determine whether significant differences exist (see Additional file 1: Appendix C for detailed test results).

In addition to the visual comparison of the observed patterns of the predicted and empirically measured effects in the monocultures and communities, we also calculated the model adequacy and model reliability according to Scholten and van der Toll [17]. We calculated the area covered by the 2.5th and 97.5th percentile of the modeled data (*M*, Eq. 4) on the one hand and of the empirical data (*O*, Eq. 5) on the other hand. In addition, we calculated the area of the intersection of *M* and *O* (*I*, Eq. 6).

4 $$M = \mathop \sum \limits_{t = 1}^{3} \left| {2.5{\text{th}}\;{\text{percentile}}\;{\text{modeled}}\;{\text{shoot}}\;{\text{mass}}_{2t} - 97.5{\text{th}}\;{\text{percentile}}\;{\text{modeled}}\;{\text{shoot}}\;{\text{mass}}_{2t} } \right|$$

5 $$O = \mathop \sum \limits_{t = 1}^{3} \left| {\hbox{min} \left( {{\text{experimental}}\;{\text{shoot}}\;{\text{mass}}} \right)_{2t} - \hbox{max} \left( {{\text{experimental}}\;{\text{shoot}}\;{\text{mass}}} \right)_{2t} } \right|$$

6 $$I = \mathop \sum \limits_{t = 1}^{3} \left| {\hbox{max} \left( {2.5{\text{th}}\;{\text{percentile}}\;{\text{modeled}}\;{\text{shoot}}\;{\text{mass}}_{2t} ,\hbox{min} \left( {{\text{experimental}}\;{\text{shoot}}\;{\text{mass}}} \right)_{2t} } \right) - { \hbox{min} }\left( {97.5{\text{th}}\;{\text{percentile}}\;{\text{modeled}}\;{\text{shoot}}\;{\text{mass}}_{2t} ,\;\hbox{max} \left( {{\text{experimental}}\;{\text{shoot}}\;{\text{mass}}} \right)_{2t} } \right)} \right|$$

Model adequacy describes which part of the experimental data can be explained by the model. It is calculated by dividing the intersection *I* with the area of the observed data in the experiments (*O*) (adequacy = *I*/*O*). Model reliability describes which part of the modeled data can also be observed in the experimental data. Therefore, we put the intersection I in relation to the modeled data area *M* (reliability = *I*/*M*). Both endpoints can have values between 0 and 1. If model adequacy is 1, all observed data fall within the range of the modeled data. If model reliability is 1, all modeled data fall within the range of the observed data. Thus, in the best case that both values are 1, there is a complete overlap between modeled and observed data and the model is able to fully represent the empirically observed data. If both the adequacy and reliability have values close to zero, there is almost no overlap between modeled and observed data (i.e., the intersection area I is close to zero).

#### Calibration results

##### Control growth

**Fig. 2:**
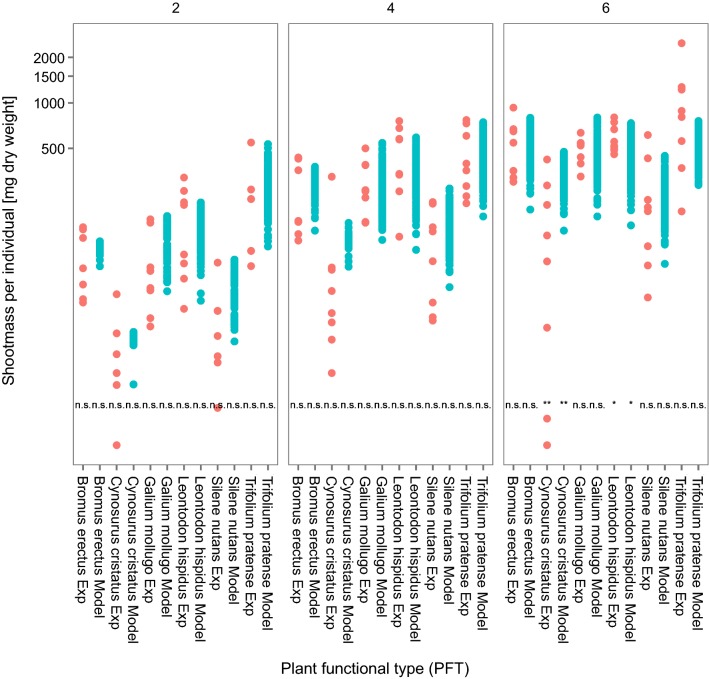
Model calibration to empirical monocultures: Comparing the shoot weight of experimentally measured (red points, *N* = 8) and modeled (blue points, *N* = 900 covering potential variability in resource levels) data for each assessment date in control monocultures. Experimental values measured in fresh weight were converted to dry weight using a conversion factor based on a repeated monoculture control experiment, in which we measured the fresh to dry weight ratio of each species (see Additional file 1: Appendix B for further details). Both broad spectrum herbicide and selective herbicide control values are included in the experimental data. Each experiment model pair was tested for significance using a *t* test; not significant results indicate that there are no differences between experimental and model data. Please note the logarithmic scale of the *y*-axis

With the model adjustments mentioned before, we were able to simulate similar biomasses in the control monocultures of the six tested plant species over the 6 weeks (Fig. 2) compared to the empirical data. Only for the last measurement in week 6, *C. cristatus* and *L. hispidus* show significant differences. In the experiments, the biomass of *C. cristatus* even decreased without any herbicide effect. The latter might imply an empirical bias, i.e., the growing conditions in the experiment were not suitable for *C. cristatus* and thus plants were impaired in their growth even without any herbicide impact. On the other hand, as we converted the empirically measured fresh weight to dry weight using a species-specific static conversion factor, the potential intraspecific and temporal variability of the fresh to dry weight ratio might be underestimated. Still, there is a high overlap between model and empirical data and on average the simulated shoot weights for the monocultures are on the same level as the empirical effects.

##### Herbicidal effects

**Fig. 3:**
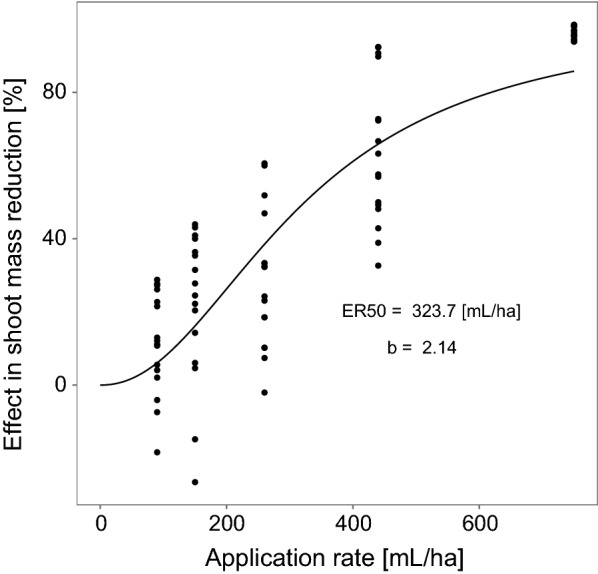
Effects on the fresh weight (% reduction of fresh weight) of *B. erectus* in the monoculture treatment 4 weeks after application, when sprayed with different application rates of the broad spectrum herbicide RoundUp^®^. Points show the empirically measured data and the line the estimated dose–response curve based on the dose-response function (Eq. 3), with the predictors for the ER50 value and the slope *b*

We calculated the dose–response function based on the empirical effects measured 4 weeks after application in the monoculture experiment. Table 4 summarizes the results of the optimization algorithm and Fig. 3 shows an exemplary dose response for *B. erectus* when affected by the broad spectrum herbicide RoundUp^®^ (see Additional file 1: Appendix D for all dose–response curves). Especially the dose responses of the selective herbicide Monitor^®^ show the different herbicide sensitivities of the test species.

**Table 4:** Estimated ER50 values and slopes b for the 6 test species and the two herbicides including the standard errors (see Additional file 1: Appendix D for all dose–response curves)

Herbicide	Species	ER50	ER50 error	Slope *b*	Slope *b* error
Broad spectrum herbicide RoundUp^®^	*B. erectus*	323.69	12.50	2.14	0.18
	*C. cristatus*	94.88	5.88	2.07	0.31
	*G. mollugo*	104.88	1.62	4.57	0.33
	*L. hispidus*	111.16	4.47	2.24	0.22
	*S. nutans*	149.93	12.09	1.46	0.18
	*T. pratense*	233.5	12.33	1.36	0.11
Selective herbicide Monitor^®^	*B. erectus*	25.23	7.20	0.64	0.12
	*C. cristatus*	1.73	0.44	0.67	0.17
	*G. mollugo*	1.52	0.06	1.93	0.19
	*L. hispidus*	1.80	0.21	0.86	0.11
	*S. nutans*	1.68	0.21	0.69	0.08
	*T. pratense*	4.74	0^a^	− 0.08	0^a^

^a^The optimization routine was not able to calculate the Hessian matrix. Therefore, we were not able to calculate an error

## Results

### Prediction of control growth in communities

**Fig. 4:**
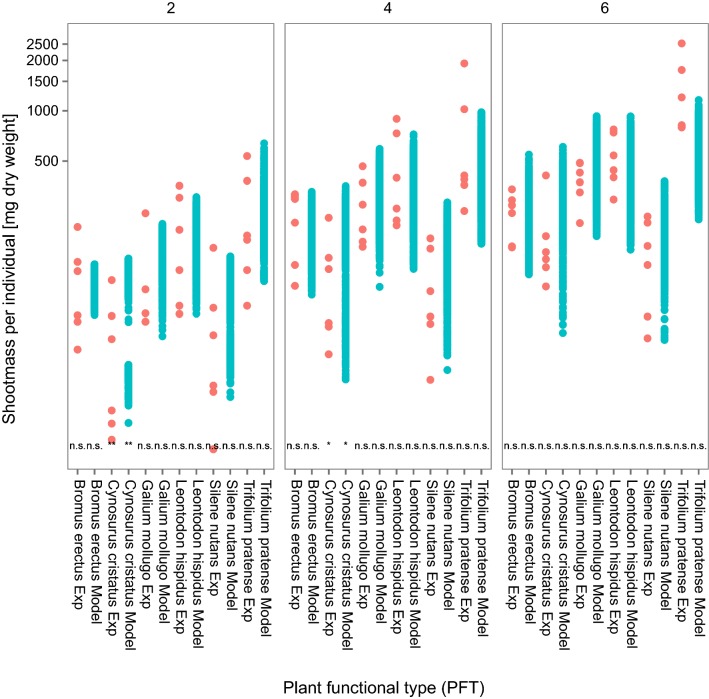
Prediction of shoot growth in control community: Comparing the shoot weight of experimentally measured (red points, *N* = 8) and modeled (blue points, *N* = 900 covering potential variability in resource levels) data for each assessment date in control communities. Experimental values measured in fresh weight were converted to dry weight using a conversion factor based on a repeated monoculture control experiment, in which we measured the fresh to dry weight ratio of each species. Both broad spectrum herbicide and selective herbicide control values are included in the experimental data. Each experiment-model pair was tested for significance using a *t*-test; not significant results indicate that there are no differences between experimental and model data. Please note the logarithmic scale of the *y*-axis

After calibration, the IBC-grass model was able to predict similar shoot masses in the artificial communities without herbicide effect (i.e., control data, Fig. 4). Most of the species-specific comparisons were not significantly different from each other. In the case of *C. cristatus*, the predicted and observed biomasses were significantly different; however, all empirical data are within the range of the model predictions. The good prediction of the control communities is underlined also by high adequacy and reliability values (Table 5). All values are above 0.6, except for the model adequacy of *T. pratense*. For this species, only 20% of the modeled shoot masses in the artificial communities are similar to empirically measured values. Nevertheless, the reliability for modeling *T. pratense* in these artificial communities is still 0.6, meaning that 60% of the observed data were predicted by the model.

**Table 5:** Model adequacy and reliability values for the predicted control communities

PFT	Adequacy	Reliability
*B. erectus*	0.67	0.89
*C. cristatus*	0.79	0.73
*G. mollugo*	0.82	0.75
*L. hispidus*	0.62	0.89
*S. nutans*	0.66	0.79
*T. pratense*	0.20	0.60

The values show the mean over all three measurements (weeks 2, 4 and 6). Model adequacy is the intersection of the modeled and empirical data space divided with the observed data space. If the value is 1, all observed data points fall within the modeled area. Model reliability is the intersection divided with the modeled data space. If the value is 1, all modeled data points fall within the observed area

### Prediction of herbicide impacts based on monoculture dose responses

#### Monocultures

**Fig. 5:**
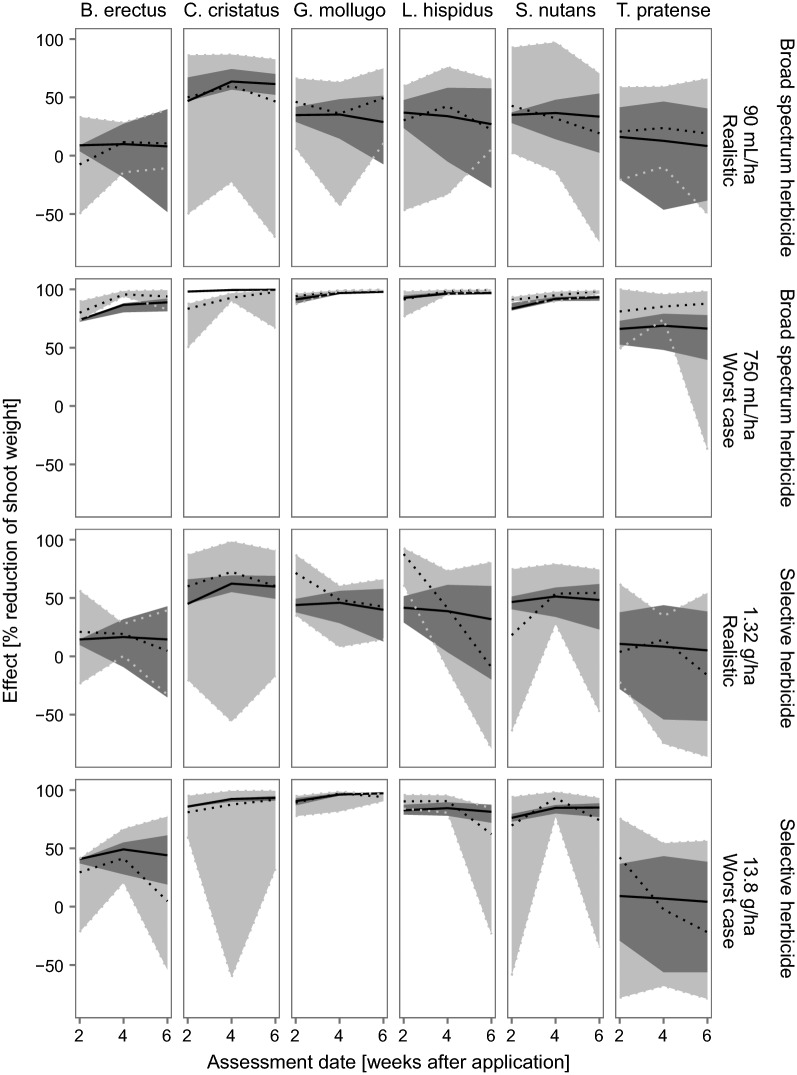
Effects on species specific shoot masses (treatment/control) in the monocultures after herbicide application. Black solid lines represent the median of the model predictions and dark gray ribbons show the upper and lower 2.5th percentile of the predictions. Dotted black lines show the empirically measured median and gray ribbons and dotted gray lines the upper and lower 2.5th percentile of these. Only the lowest (realistic) and highest (worst-case) tested application rates are presented here. The lowest rates tested were in the same range (broad spectrum herbicide Roundup) or higher (selective herbicide Monitor) than the rates that would be applicable to the risk assessment considering European standard drift rates. The results for these test rates are presented to deliver a more realistic picture. Results for the whole range of tested application rates can be found in Additional file 1: Appendix E

In the monoculture treatment, the calibrated IBC-grass model showed a good reliability for both the selective herbicide as well as the broad spectrum herbicide (Table 6). In 56–100% of the simulation runs, in which we varied above and belowground resource levels (see Methods, overall 900 simulation runs with 90 different resource level combinations and 10 repetitions for each combination), the predicted effects are within the range of the experimentally measured effects (Fig. 5). The observed effects on the shoot masses of the 6 different PFTs over time (i.e., patterns) are comparable to the patterns predicted by the model. For example, for the realistic drift rate of 3.0% of the maximum application rate of the broad spectrum herbicide RoundUp^®^ (≡ 90 mL/ha), the mean effect on the shoot mass of *B. erectus* remained negligible in both the empirical data as well as in the model predictions. In contrast to that, the mean effects on *C. cristatus* are increasing over time in both the empirical and modeled data. However, especially at this lowest test rate which is similar or slightly higher than the predicted EU drift rate (2.77%), some species show a very high variation in the experiments (e.g., *C. cristatus*). That biological variation is not covered in IBC-grass, which is also reflected in lower model adequacy (Table 6). The design of the toxicological submodel, transferring the empirical effects measured in the monocultures 4 weeks after application as a weekly reduction in the biomass gain, results in a good representation of the observed patterns and temporal dynamics of the species-specific effects. Nevertheless, it needs to be kept in mind that the dose responses, on which the individual-level effects are based on in the model, were calculated using the empirical monoculture data 4 weeks after application. Thus, the modeled data are not completely independent from the empirical data.

**Table 6:** Model fit for the monocultures

PFT	Selective herbicide		Broad spectrum herbicide	
	Adequacy	Reliability	Adequacy	Reliability
*B. erectus*	0.43	0.89	0.39	0.73
*C. cristatus*	0.08	1.00	0.04	0.56
*G. mollugo*	0.25	0.91	0.32	0.74
*L. hispidus*	0.35	0.75	0.32	0.83
*S. nutans*	0.17	0.98	0.20	0.73
*T. pratense*	0.72	0.98	0.56	0.84
All	0.33	0.92	0.31	0.74

Mean model adequacy and reliability over all herbicide application rates for the selective and the broad spectrum herbicide. Model adequacy is the intersection of the modeled and empirical data space divided with the observed data space. If the value is 1, all observed data points fall within the modeled area. Model reliability is the intersection divided with the modeled data space. If the value is 1, all modeled data points fall within the observed area

#### Artificial communities

**Fig. 6:**
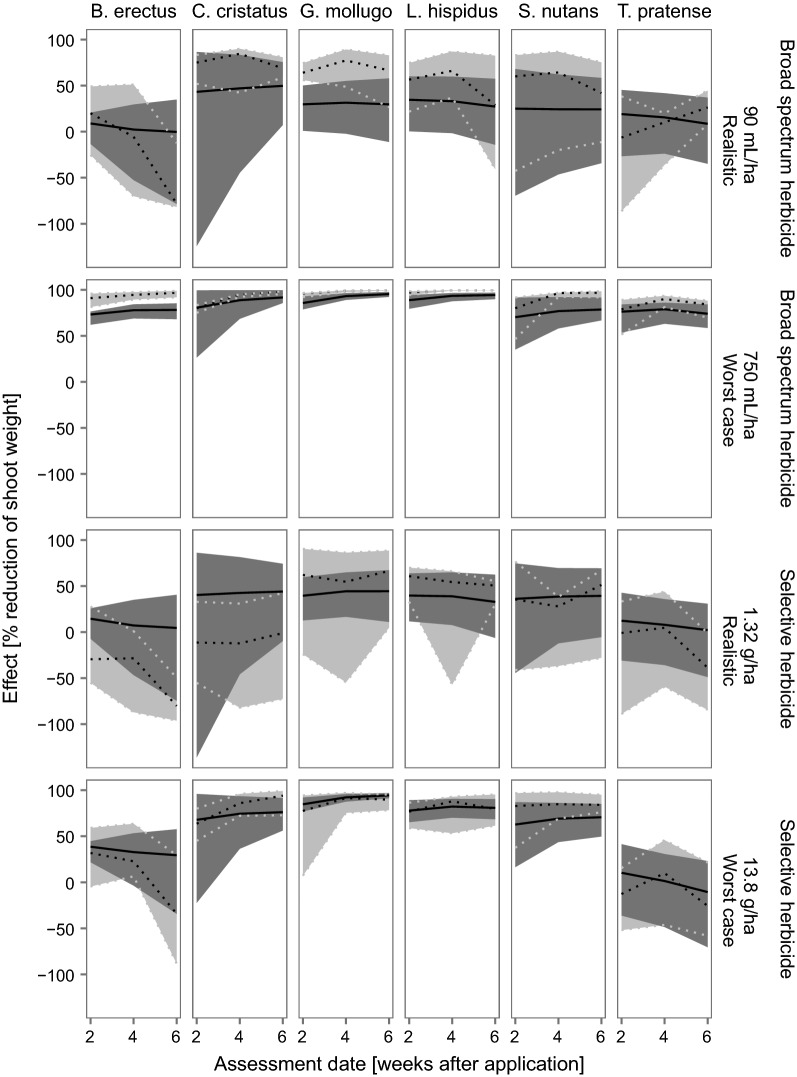
Effects on species specific shoot masses (treatment/control) in the artificial communities after herbicide application. Black solid lines represent the median of the model predictions and dark gray ribbons show the upper and lower 2.5th percentile of the predictions. Dotted black lines show the empirically measured median and gray ribbons and dotted gray lines the upper and lower 2.5th percentile of these. Only the lowest (realistic) and highest (worst-case) tested application rates are presented here. The lowest rates tested were in the same range (broad spectrum herbicide RoundUp^®^) or higher (selective herbicide Monitor^®^) than the rates that would be applicable to the risk assessment considering European standard drift rates. The results for these test rates are presented to deliver a more realistic picture. Results for the whole range of tested application rates can be found in Additional file 1: Appendix E

In general, the predicted temporal patterns and magnitude of the effects on plant populations in an artificial community of the model IBC-grass are comparable to the observed patterns and magnitude (Fig. 6). Model adequacy is higher than in the monocultures (Table 7, compared to Table 6). The variation in the simulated communities is greater than that in the monocultures due to the additional interspecific competition. Therefore, the model is able to cover the natural variability found in the experiments to a greater extent. There are slight differences between the two herbicides (selective and broad spectrum herbicide), but looking at all plant species (or PFTs), the adequacy is the same. Model reliability is smaller than in the monocultures, especially for the broad spectrum herbicide, meaning that a lower percentage of the model predictions is within the range of observed effects (Fig. 6). This can be explained by a higher variability in the predicted effects due to interspecific competition between plant individuals (compared to the monocultures).

**Table 7:** Model fit for the communities

PFT	Selective herbicide		Broad spectrum herbicide	
	Adequacy	Reliability	Adequacy	Reliability
*B. erectus*	0.46	0.53	0.48	0.63
*C. cristatus*	0.73	0.52	0.93	0.17
*G. mollugo*	0.37	0.93	0.41	0.28
*L. hispidus*	0.55	0.78	0.49	0.43
*S. nutans*	0.79	0.74	0.67	0.42
*T. pratense*	0.77	0.86	0.65	0.73
All	0.61	0.73	0.61	0.44

Mean model adequacy and reliability over all herbicide application rates of the selective (Monitor^®^) and the broad spectrum herbicide (RoundUp^®^). Model adequacy is the intersection of the modeled and empirical data space divided with the observed data space. If the value is 1, all observed data points fall within the modeled area. Model reliability is the intersection divided with the modeled data space. If the value is 1, all modeled data points fall within the observed area

## Discussion

In a plant community, inter- and intraspecific competition plays a major role in determining the dynamics within the community. Thus, indirect impacts of herbicides on populations as well as on plant community dynamics might appear, especially if affected plants differ in their susceptibility (e.g., if selective herbicides are applied). As a result, the competitive relationship between plant individuals in a community might shift between individuals of different plant species and, consequently, indirect impacts might alter plant community dynamics (see, e.g., [21]). Such indirect effects on plant species not impaired by the herbicide were observed in the study of Reuter and Siemoneit-Gast [12], especially regarding the selective herbicide Monitor^®^. Already in this short-term study, for instance the shoot mass of *T. pratense*, being less sensitive to the herbicide than other test species, increased due to lower interspecific competition from more sensitive plant species such as *G. mollugo* with a decreasing shoot mass. After the calibration process, where the IBC-grass model was solely adapted to the monoculture control data of the experiment [12], the model was able to not only predict shoot masses measured in control communities without further model adaptations, but also to predict similar effect intensities and dynamics over time in these artificial communities. Similar to the empirical results, *T. pratense* showed only minor decreases in shoot masses or even an increase under herbicide treatments; whereas for *G. mollugo* the model predicted a high negative impact on the shoot mass.

The study setup allows us to make conclusions about the intra- as well as interspecific competition and their reflection in the model: in monocultures, only plant individuals of the same species compete for resources and space, whereas in the artificial communities both types of competition occur: between individuals of the same species and between individuals of different species. Although we did not quantify the specific impact of intra- or interspecific competition in the empirical data, it can be assumed that competition occurs as soon as plant individuals overlap in their roots or shoots. As the distance between the plant individuals is quite small (2.5 cm), an overlap of roots and shoots is very likely. We were able to calibrate the model to the monoculture control growth, where only intraspecific competition took place. This allows the conclusion that the intraspecific competition is indeed well reflected in the model. The fact that we did not touch any process regarding the competition during the calibration process even strengthens this conclusion. Excluding the competition from this calibrated model actually showed that simulated plant growth would have exceeded the empirical measurements (Additional file 1: Appendix F). Also interspecific competition is well reflected in the model: On the one hand, the model predicted similar shoot masses in control communities. The main difference compared to the monoculture simulation is actually the interspecific competition process. And, on the other hand, also similar effect intensities and temporal dynamics under herbicide treatment were predicted. Thus, indirect effects resulting from intraspecific competition due to the different species specific susceptibilities are reflected by the model.

Furthermore, the model was able to predict similar short-term herbicide impacts on communities based on the species specific dose responses (calculated from empirical effects in monocultures measured 4 weeks after application) compared to empirical data. The guidance document currently in use in the EU specifies an assessment factor, which is supposed to cover the uncertainties in the risk assessment for non-target terrestrial plants, e.g., the extrapolation from individual-level tests to the community level or the occurrence of even more sensitive species [38]. However, the appropriateness of the assessment factor for covering uncertainties is debated. Participants of a non-target terrestrial plant workshops held by the Society of Environmental Toxicology and Chemistry (SETAC) in 2014 and 2015 recommended using modeling approaches to support the risk assessment of terrestrial plant communities [39]. The current study strengthens that the presented plant community model IBC-grass is suitable to be used for analyzing short-term effects on a plant community based on monoculture dose responses, which can be derived from the standard studies used for the current non-target terrestrial plant risk assessment. However, also the extrapolation from short-term to long-term effects is important to estimate the potential risk on non-target terrestrial plant communities. Therefore, longer term field data are required to evaluate the models’ accuracy and reliability for predicting long-term impacts of herbicides and therewith strengthen the model’s credibility for risk assessors. It would be valuable if new empirical studies were designed to be used as additional data for validating ecological models like IBC-grass, e.g., measuring biomass on individual level over a longer time period.

Plant communities show a high natural variability. This variability is caused by various factors, e.g., heterogeneity in the soil (i.e., in nutrients or moisture) or aboveground and belowground disturbances by grazing, trampling or management practices, but also the history of the landscape is important for its current state. To adequately characterize herbicide-related effects on plant communities, a high amount of replication is needed. Thus, the field studies are not only labor and cost intensive but also put high demands on the study site, e.g., a large homogenous field area in order to disentangle the herbicide impact from other factors determining the variability. Ecological models can overcome this dilemma if they comprise the main drivers for the variability in plant community dynamics, which were mentioned earlier. Different scenarios (e.g., resource levels, management practices) can be simulated to cover for various conditions occurring in semi-natural grasslands. In IBC-grass indeed many of these factors are included: resource levels and disturbances such as grazing, trampling and cutting are integrated in the model. As previous studies showed, the IBC-grass model is able to predict also long-term impacts on grassland communities. For example, Weiß et al. [19] analyzed the effect of different grazing intensities and realistically predicted the yield under different grazing regimes. Integrating resource heterogeneity directly in the model might be desirable for improving the model performance. However, heterogeneity in resources can also be covered by simulating small plots with a variety of potential resource levels similar to sample subplots in empirical studies to cover the natural variability, which is comparable to the approach in the current study.

As several environmental parameters (e.g., resource levels, disturbances, PFT pool) can be changed in IBC-grass, different environments can be covered. However, the model was originally developed and validated for semi-natural grasslands in Germany. Therefore, special environmental conditions, e.g., occurring in drylands or wetlands, which are driven or limited by other factors such as soil moisture, fire or salinity, are currently not covered in the model. Thus, the processes driving these specific ecosystems would need to be integrated in the model beforehand in order to be suitable for the corresponding risk assessments. However, for semi-natural grasslands in regions, which have similar environmental conditions to German grasslands, IBC-grass can provide reasonable assessments of potential outcomes of herbicide impacts on community level (see [21] for potential long-term effects of herbicide impacts on different grassland communities occurring adjacent to agricultural fields in Germany).

To evaluate the credibility of model predictions using empirical data, we chose to calculate model adequacy and reliability [17]. Both values are equally important to qualify the model predictions. Reliability explains which part of the model predictions is observed in the empirical data. Thus, it is a measure to estimate in how many simulations the model is in agreement with empirical data. Adequacy, on the other hand explains which part of the observed data is predicted. It gives an idea whether the model is covering also the extreme cases, e.g., the strongest effects that were observed. Therefore, both measurements should always be reported and considered in combination. For example, if all model predictions have also been observed, but only cover a small range of the variability in the observed data, the reliability is high; however, the adequacy is low. Ideally, you would want to have a high adequacy and a high reliability. In general, it is important to be aware of the instances which might not be covered by the model.

With the detailed model description following the ODD protocol [21], sensitivity analyses [19, 22] and the short-term validation in the current study, IBC-grass now fulfills the main aspects for an ecological model to be used for ecological risk assessments and thus for environmental decision making [40]. Based on individual-level effects measured in standard greenhouse experiments, IBC-grass can extrapolate the effects up to community level. Thus, a range of different environmental scenarios and the effect on different grassland communities can be simulated to estimate the potential risk posed by herbicide applications on non-target terrestrial plants. The current study showed that for short-term effects the model is realistically predicting the community-level effects. To strengthen the credibility of the model also for long-term effects, a validation based on long-term effect data is desirable; however, it is difficult to reach as there is a lack of suitable long-term field studies.

## Conclusions

In this current study, we were able to show that the plant community model IBC-grass was able to realistically predict short-term community-level effects on plant biomass based on monoculture dose–response data. It represents an approach how individual-level effects measured in current standard greenhouse experiments can be integrated in a community model to estimate community-level effects in ecological risk assessments of herbicides. Such validated plant community models might be especially important in the future as EFSA considers specific protection goals for non-target terrestrial plants on population and community level [2].

## Additional file

**Additional file 1.** Supporting information.

## Abbreviations

EFSA: European food safety authority
OECD: Organisation for Economic Co-operation and Development
IBC-grass: individual-based plant community model for grasslands
PFT: plant functional type
ODD: overview, design concepts, details
ZOI: zone of influence
BSH: broad spectrum herbicide
SH: selective herbicide
