# Supplementary material for: Simulation of herbicide impacts on a plant community: comparing model predictions of the plant community model IBC-grass to empirical data
Source: Environ Sci Eur. 2018 Nov 14;30(1):44. doi: 10.1186/s12302-018-0174-9 (PMC6244561; doi:10.1186/s12302-018-0174-9)
Supplement: Supplementary file 1 — Additional file 1. Supporting information. [file 12302_2018_174_MOESM1_ESM.docx]

## Appendix A: Model settings and parameters

Table A.1: Parameters for the environmental setttings

| **Variable** | **Explanation** | **Unit** | **Value** |
| --- | --- | --- | --- |
| Plot size |  | cm | 7x7; 20x20 |
| Aboveground resources |  | resource units/cm² | 50-100 |
| Belowground resources |  | resource units/cm² | 60-120 |
| Grazing | Grazing probability |  | 0 |
| Trampling | Trampling probability |  | 0 |
| Cutting | Cutting events |  | 0 |
| Tmax | Maximal time simulated | year | 1 |
| Seed input |  | Nb of seeds/year | 0 |
| pbase | Base mortality | % | 0 |
|  |  |  |  |

Table A.2: PFT specific parameters

| **Variable** | **Explanation** | **Unit** | **PFT/species** | | | | | | |
| --- | --- | --- | --- | --- | --- | --- | --- | --- | --- |
|  |  |  | *B. erectus* | *C.*  *cristatus* | *G. mollugo* | *L. hispidus* | *S. nutans* | *T. pratense* | |
| fleaf | Growth form | | 0.75 | 0.75 | 1.0 | 0.5 | 0.75 | 0.75 | |
| mmax | Maximal plant mass | mg dry weight | 5000 | 5000 | 2000 | 2000 | 2000 | 2000 | |
| mseed | Seed mass | mg dry weight | 1.0 | 1.0 | 0.3 | 0.3 | 0.3 | 0.3 | |
| meandisp | Mean dispersal distance | m | 0.1 | 0.1 | 0.3 | 0.3 | 0.3 | 0.3 | |
| stddisp | Standard deviation of dispersal distance | m | 0.1 | 0.1 | 0.3 | 0.3 | 0.3 | 0.3 | |
| Gmax | Maximal resource utilization per time step and ZOI area (equal for shoot and root) | resource units/cm² | 20 | 40 | 60 | 40 | 40 | 60 | |
| survmax | Maximal survival time during resource stress | weeks | 6 | 4 | 2 | 4 | 4 | 2 | |
| palatability | Susceptibility towards grazing | | 1.0 | 0.25 | 0.5 | 1.0 | 0.5 | 1.0 | |
| cshoot | Above-ground ZOI area per leaf mass | cm²/mg | 1.0 | 0.5 | 0.75 | 1.0 | 0.75 | 1.0 | |
| RAR | Belowground ZOI area per root mass | cm²/mg | 1.0 | 1.0 | 1.0 | 1.0 | 1.0 | 1.0 | |
| growth | Conversion rate resource to biomass | mg/  resource unit | 0.25 | | | | | | |
| mThres | Threshold of Δ_resmax for resource stress | | 0.2 | | | | | | |
| Resshare | Resource sharing between ramets of the same individual | | 0 | | | | | | |
| SpacerL | Spacer length | cm | 0 | | | | | | |
| stdSpacerL | Standard deviation of spacer length | cm | 0 | | | | | | |
| mSpacer | resources for 1 cm spacer (default=70) | resource unit/cm | 0 | | | | | | |
| Alloc_root | Factor for increased allocation into roots | | 1.0 | 1.0 | 1.0 | 1.0 | 0.5 | | 1.0 |
| Alloc_shoot | Factor for increased allocation into shoots | | 1.0 | 1.0 | 1.0 | 1.0 | 1.0 | | 0.2 |

## Appendix B: Repetition of the experimental monoculture control scenario

Since the model IBC-grass calculates plant growth based on the dry weight but Reuter and Siemoneit-Gast measured only fresh weight, we repeated the monoculture control scenario in order to calculate a dry to fresh weight ratio per plant species which we could then apply to all experimental data. The study was conducted in the greenhouse of the Botanical Garden in Potsdam, Germany, from March to May 2017.

### Seedling Cultivation

The six plant species were cultivated on palettes with 240 cells (2.3x2.3x3.7 cm). The cells were filled with standard cultivation soil. At the beginning, several seeds were sown per cell to guarantee that at least one seed per cell germinated. If more than one seed germinated, all except for the one most in the middle were removed after emergence. After seeding, the palettes were put on a seed culture pot of 53x31x5.5 cm with a standard irrigation mat at the bottom of the pot. After initial watering from below to water the fleece mat, the palettes were watered on demand from above. At the beginning, a cold frame fleece was put on top of the palettes in order to support germination. It was removed during the main germination period. For each plant species, we seeded 160 cells, with the goal to have at least 120 emerging individuals, leading to 10 replicates per assessment date (3 assessment dates, 4 individuals per pot).

### Culture conditions

When most seedlings reached the two to four leaf stage (BBCH 12-14), plant individuals were transplanted to the monoculture set up: 4 plant individuals of similar height were transplanted (homogenously distributed) into one pot (7x7x5 cm, filled with medium loamy soil and a deposit fertilizer). Plants were watered from above on demand.

The emergence rate differed strongly between the plant species. E.g. for *Bromus erectus* very few individuals emerged. Thus, the number of pots/replicates per species was different (Table A.1).

Table B.1: Number of replicates per plant species after transplantation. One replication represents one pot with 4 individuals.

| **Assessment date** | ***B. erectus*** | ***C. cristatus*** | ***G. mollugo*** | ***L. hispidus*** | ***S. nutans*** | ***T. pratense*** |
| --- | --- | --- | --- | --- | --- | --- |
| 2 | 3 | 10 | 9 | 4 | 8 | 8 |
| 4 | 3 | 10 | 9 | 4 | 8 | 9 |
| 6 | 4 | 10 | 9 | 4 | 8 | 9 |
| **Sum** | **10** | **30** | **27** | **12** | **24** | **26** |

### Measurements

Aboveground biomass was harvested from a fraction of the replicates 2, 4 and 6 weeks after transplantation (see Table B.1). During the experiment, some plants died. Only pots with at least one living plant were included in the assessment. Pots with only dead plants were ignored and therefore, the number of replicates decreased for some plant species (namely *C. cristatus, L. hispidus and T. pratense,* see Tables B.1 vs. B.2). However, at least 3 pots/replicates were measured per assessment date and species. Fresh weight per pot was measured directly after harvesting (excluding dead plants). The plants were dried for 3 days at 60 degrees Celsius to measure the dry weight. Afterwards, the ratio of dry to fresh weight was calculated per pot/replicate.

### Results

The fresh to dry weight ratios are similar between the tested species except for *T. pratense*, which had the highest ratios (Table B.2). We used the mean of all assessment dates as a factor to convert the fresh weight measured in the study of Reuter and Siemoneit-Gast (2007) to dry weight. Based on this conversion factor, the modeled monoculture control dry weights fit to the converted dry weights of the experimental monoculture controls in the study of Reuter and Siemoneit-Gast (2007) (see Figure 2 in the main manuscript).

Table B.2: Mean dry to fresh weight ratios for the three assessment dates: 2, 4 and 6 weeks after transplantation. Numbers in brackets represent the number of replicates (i.e. the number of pots).

| **Assessment date** | ***B. erectus*** | ***C. cristatus*** | ***G. mollugo*** | ***L. hispidus*** | ***S. nutans*** | ***T. pratense*** |
| --- | --- | --- | --- | --- | --- | --- |
| 2 | 27% (3) | 21% (10) | 18% (9) | 21% (4) | 17% (8) | 20% (8) |
| 4 | 26% (3) | 33% (10) | 26% (9) | 16% (4) | 21% (8) | 52% (9) |
| 6 | 23% (4) | 25% (9) | 24% (9) | 16% (3) | 20% (8) | 32% (4) |
| **Overall mean** | **25%** | **27%** | **22%** | **18%** | **19%** | **36%** |

## Appendix C: Complete significance test results

Table C.1: Welch Two Sample t-test statistics for the model calibration (using monoculture control data).

| PFT | Time [week] | Value t-statistic | Degrees of freedom | estimate | | p-value | |
| --- | --- | --- | --- | --- | --- | --- | --- |
|  |  |  |  | model | experiment |  |  |
| Bromus erectus | 2 | 1.17 | 7 | 120.06 | 101.25 | 0.282 | n.s. |
|  | 4 | 1.19 | 6 | 321.76 | 257.86 | 0.279 | n.s. |
|  | 6 | 0.21 | 7 | 531.85 | 515.31 | 0.840 | n.s. |
| Cynosurus cristatus | 2 | 1.38 | 7 | 28.56 | 21.26 | 0.210 | n.s. |
|  | 4 | 1.70 | 7 | 142.64 | 82.35 | 0.133 | n.s. |
|  | 6 | 3.76 | 7 | 344.96 | 147.83 | 0.007 | ** |
| Galium mollugo | 2 | 1.31 | 7 | 108.79 | 84.43 | 0.231 | n.s. |
|  | 4 | 0.04 | 7 | 296.51 | 294.80 | 0.969 | n.s. |
|  | 6 | -1.14 | 7 | 452.38 | 492.25 | 0.291 | n.s. |
| Leontodon hispidus | 2 | -0.60 | 7 | 141.78 | 163.58 | 0.565 | n.s. |
|  | 4 | -1.58 | 7 | 332.29 | 456.53 | 0.157 | n.s. |
|  | 6 | -3.27 | 7 | 442.54 | 592.20 | 0.014 | * |
| Silene nutans | 2 | 1.59 | 6 | 51.08 | 35.56 | 0.162 | n.s. |
|  | 4 | 1.10 | 7 | 146.47 | 117.09 | 0.310 | n.s. |
|  | 6 | 0.03 | 7 | 240.88 | 239.16 | 0.980 | n.s. |
| Trifolium pratense | 2 | 0.90 | 6 | 278.47 | 223.71 | 0.402 | n.s. |
|  | 4 | -0.22 | 7 | 432.22 | 449.55 | 0.831 | n.s. |
|  | 6 | -1.80 | 7 | 517.25 | 973.35 | 0.115 | n.s. |

Table C.2: Welch Two Sample t-test statistics for the model prediction of community control growth

| PFT | Time [week] | Value t-statistic | Degrees of freedom | estimate | | p-value | |
| --- | --- | --- | --- | --- | --- | --- | --- |
|  |  |  |  | model | experiment |  |  |
| Bromus erectus | 2 | 0.34 | 5 | 106.91 | 98.33 | 0.746 | n.s. |
|  | 4 | -0.91 | 5 | 187.27 | 224.58 | 0.403 | n.s. |
|  | 6 | 0.27 | 5 | 251.18 | 242.92 | 0.800 | n.s. |
| Cynosurus cristatus | 2 | 4.68 | 5 | 105.08 | 40.05 | 0.005 | ** |
|  | 4 | 3.03 | 5 | 193.00 | 102.60 | 0.029 | * |
|  | 6 | 2.16 | 5 | 281.72 | 177.75 | 0.083 | n.s. |
| Galium mollugo | 2 | 1.53 | 5 | 139.61 | 92.77 | 0.187 | n.s. |
|  | 4 | 0.44 | 5 | 294.03 | 271.33 | 0.677 | n.s. |
|  | 6 | 1.11 | 5 | 433.61 | 385.73 | 0.318 | n.s. |
| Leontodon hispidus | 2 | 0.09 | 5 | 187.02 | 182.40 | 0.931 | n.s. |
|  | 4 | -0.79 | 5 | 357.91 | 452.70 | 0.464 | n.s. |
|  | 6 | -1.04 | 5 | 450.22 | 531.90 | 0.345 | n.s. |
| Silene nutans | 2 | 1.47 | 5 | 84.40 | 52.88 | 0.203 | n.s. |
|  | 4 | 1.91 | 5 | 136.15 | 90.57 | 0.115 | n.s. |
|  | 6 | 1.28 | 5 | 178.91 | 137.75 | 0.256 | n.s. |
| Trifolium pratense | 2 | -0.32 | 5 | 217.74 | 241.20 | 0.763 | n.s. |
|  | 4 | -1.01 | 5 | 458.37 | 726.00 | 0.357 | n.s. |
|  | 6 | -2.54 | 5 | 592.81 | 1318.20 | 0.052 | n.s. |

## Appendix D: Dose response curves for all species and both herbicides


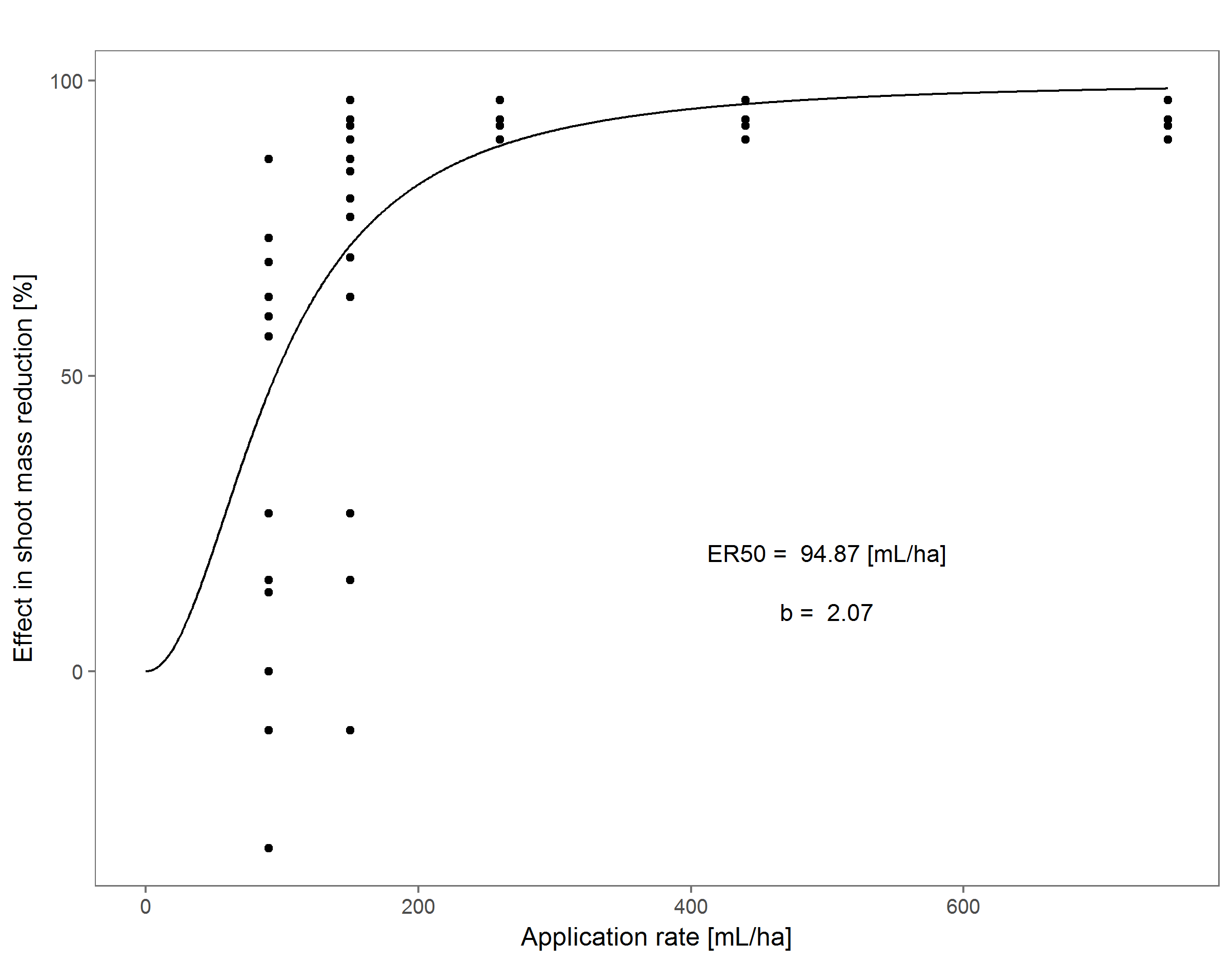


Figure D.1: Effects on the fresh weight (% reduction of fresh weight) of *C. cristatus* in the monoculture treatment 4 weeks after application, when sprayed with different application rates of the broad spectrum herbicide RoundUp®. Points show the empirically measured data and the line the estimated dose response function, with the predictors for the ER50 value and the slope b.


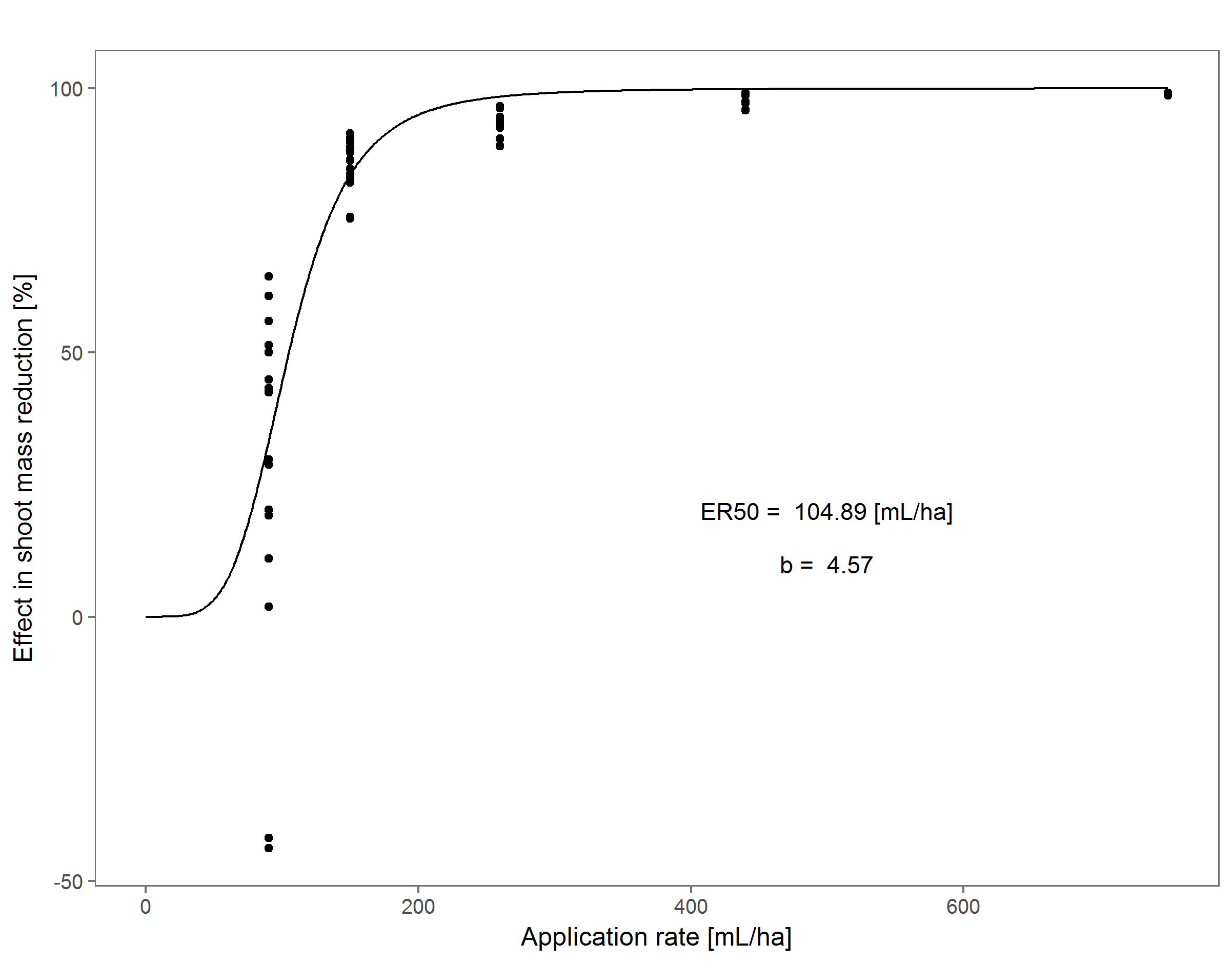


Figure D.2: Effects on the fresh weight (% reduction of fresh weight) of *G. mollugo* in the monoculture treatment 4 weeks after application, when sprayed with different application rates of the broad spectrum herbicide RoundUp®. Points show the empirically measured data and the line the estimated dose response function, with the predictors for the ER50 value and the slope b.


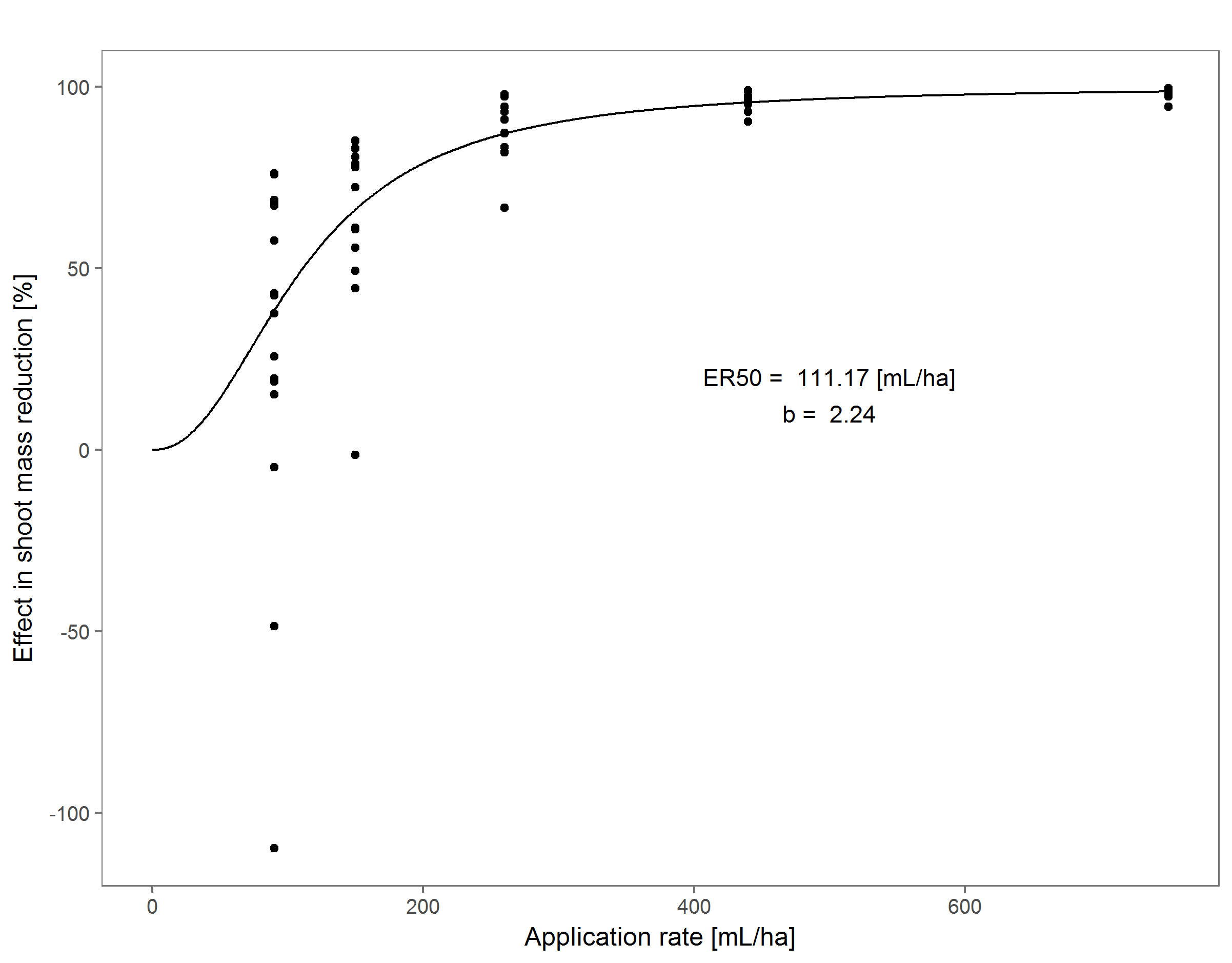


Figure D.3: Effects on the fresh weight (% reduction of fresh weight) of *L. hispidus* in the monoculture treatment 4 weeks after application, when sprayed with different application rates of the broad spectrum herbicide RoundUp®. Points show the empirically measured data and the line the estimated dose response function, with the predictors for the ER50 value and the slope b.


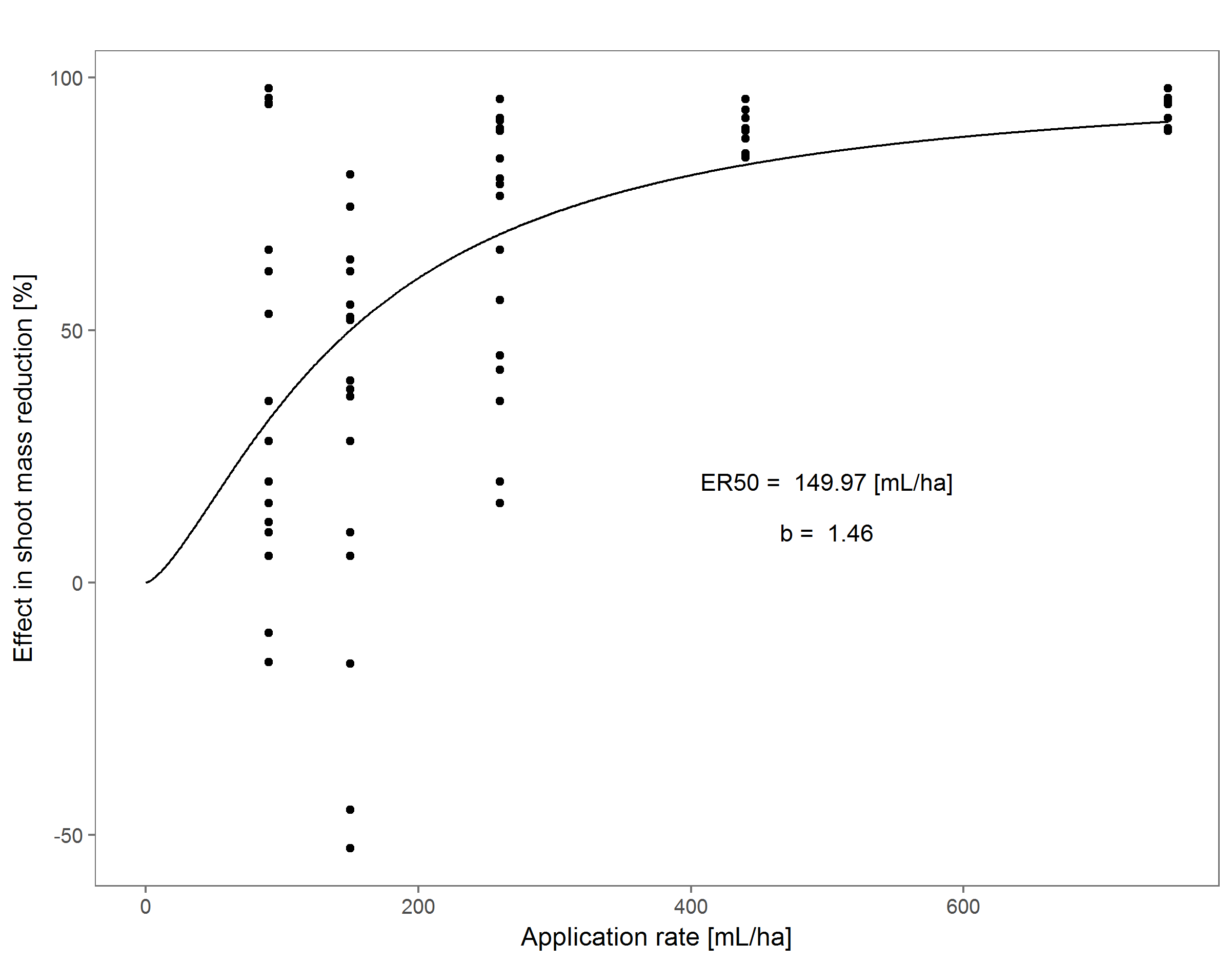


Figure D.4: Effects on the fresh weight (% reduction of fresh weight) of *S. nutans* in the monoculture treatment 4 weeks after application, when sprayed with different application rates of the broad spectrum herbicide RoundUp®. Points show the empirically measured data and the line the estimated dose response function, with the predictors for the ER50 value and the slope b.


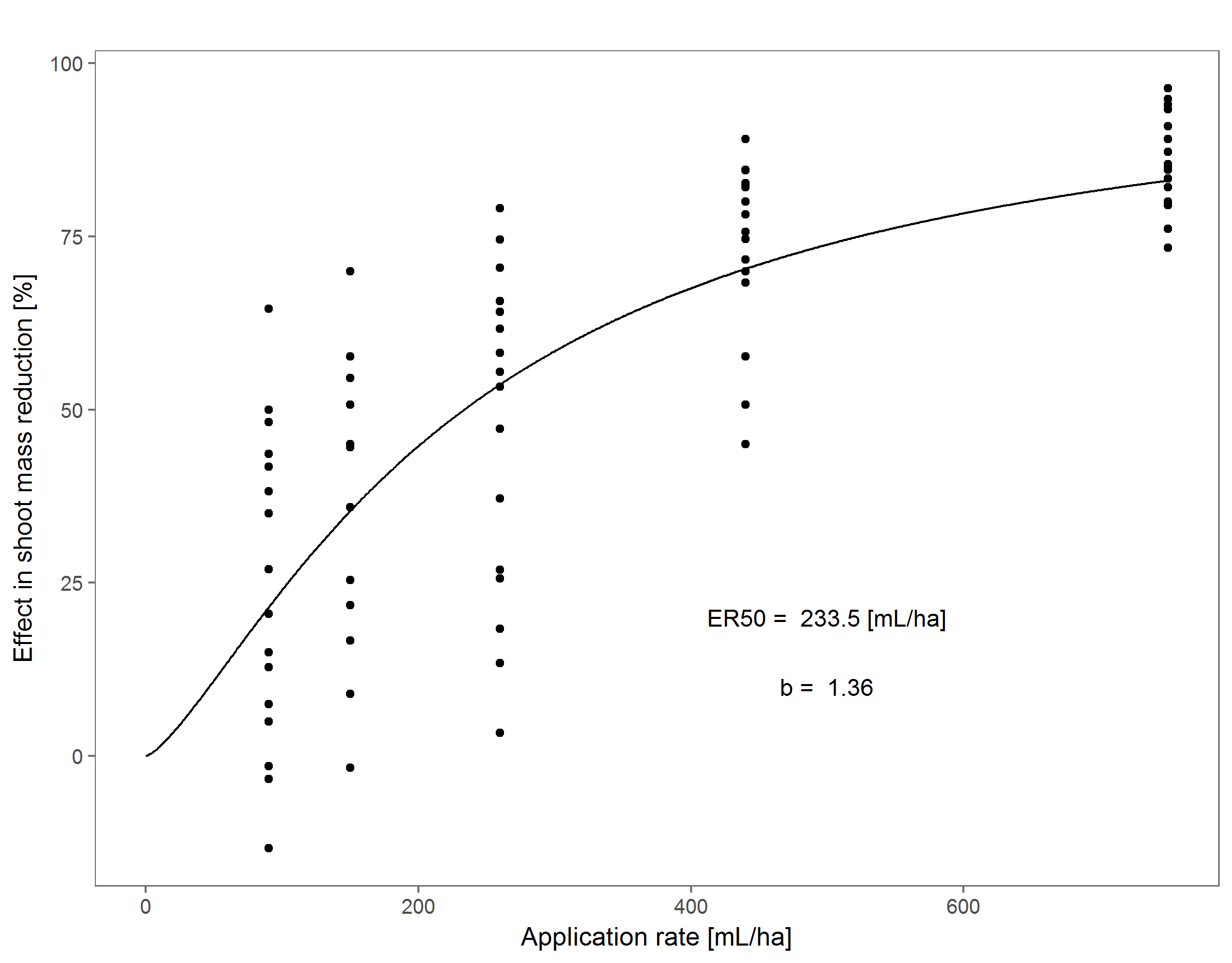


Figure D.5: Effects on the fresh weight (% reduction of fresh weight) of *T. pratense* in the monoculture treatment 4 weeks after application, when sprayed with different application rates of the broad spectrum herbicide RoundUp®. Points show the empirically measured data and the line the estimated dose response function, with the predictors for the ER50 value and the slope b.


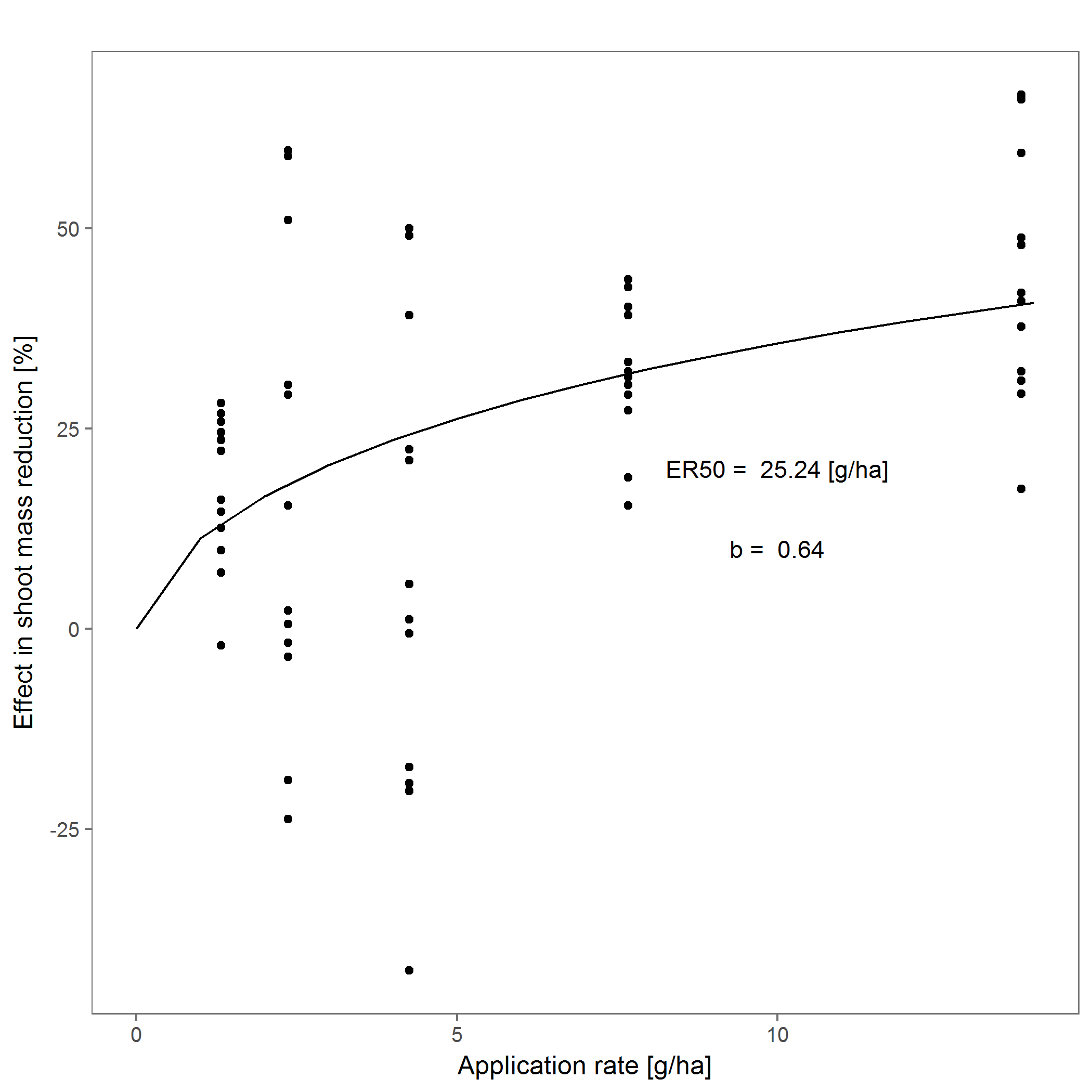


Figure D.6: Effects on the fresh weight (% reduction of fresh weight) of *B. erectus* in the monoculture treatment 4 weeks after application, when sprayed with different application rates of the selective herbicide Monitor®. Points show the empirically measured data and the line the estimated dose response function, with the predictors for the ER50 value and the slope b.


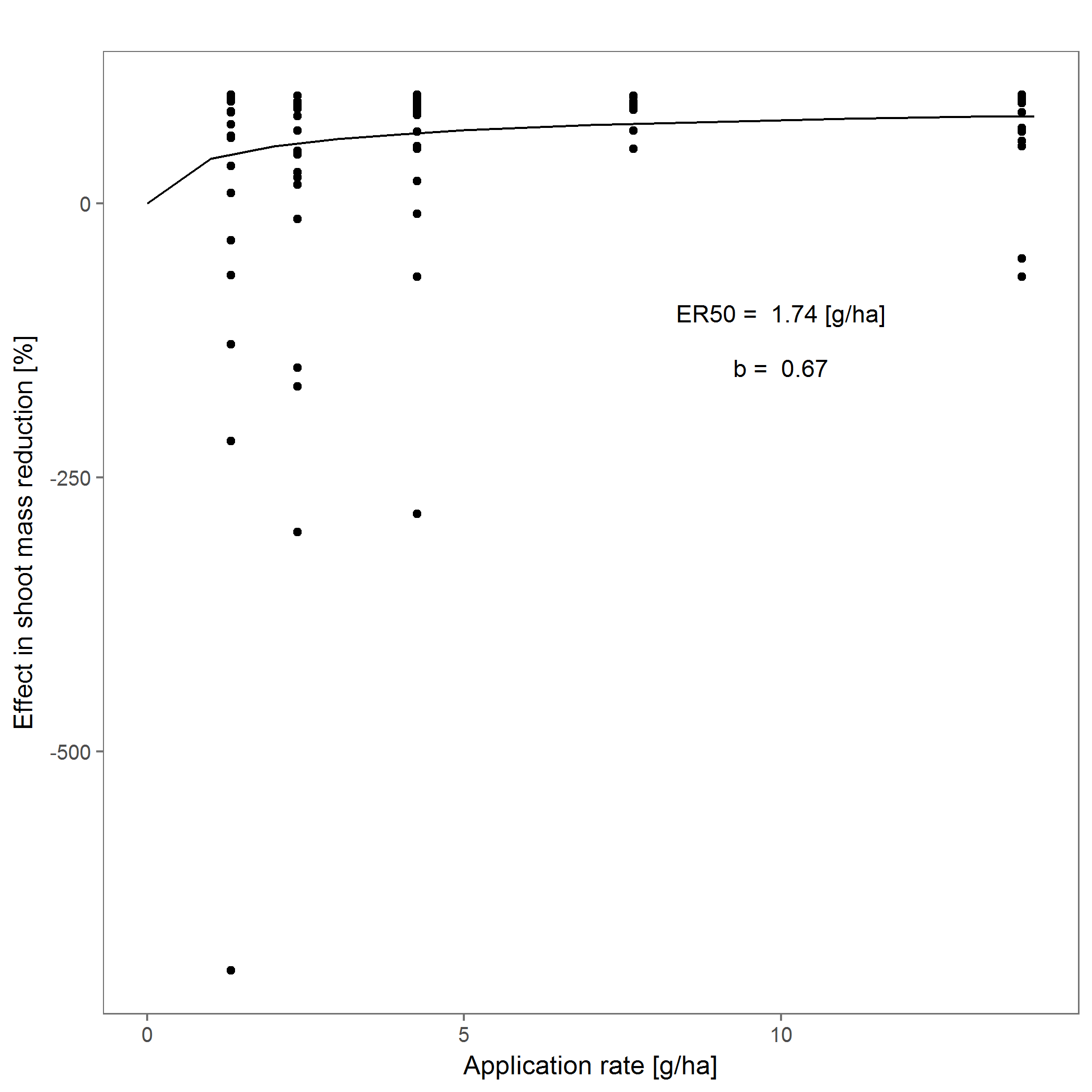


Figure D.7: Effects on the fresh weight (% reduction of fresh weight) of *C. cristatus* in the monoculture treatment 4 weeks after application, when sprayed with different application rates of the selective herbicide Monitor®. Points show the empirically measured data and the line the estimated dose response function, with the predictors for the ER50 value and the slope b.

**
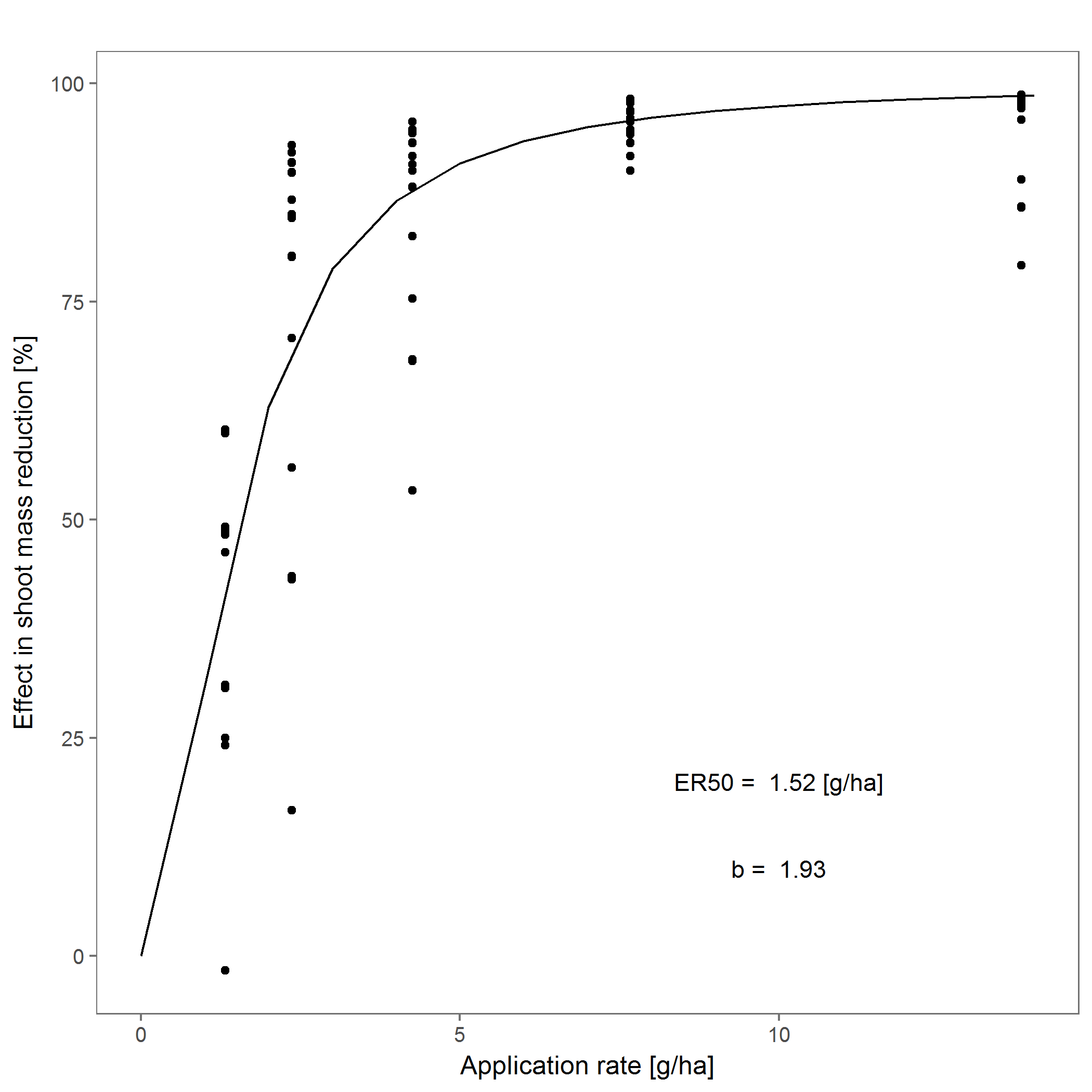
**

Figure D.8: Effects on the fresh weight (% reduction of fresh weight) of *G. mollugo* in the monoculture treatment 4 weeks after application, when sprayed with different application rates of the selective herbicide Monitor®. Points show the empirically measured data and the line the estimated dose response function, with the predictors for the ER50 value and the slope b.

**
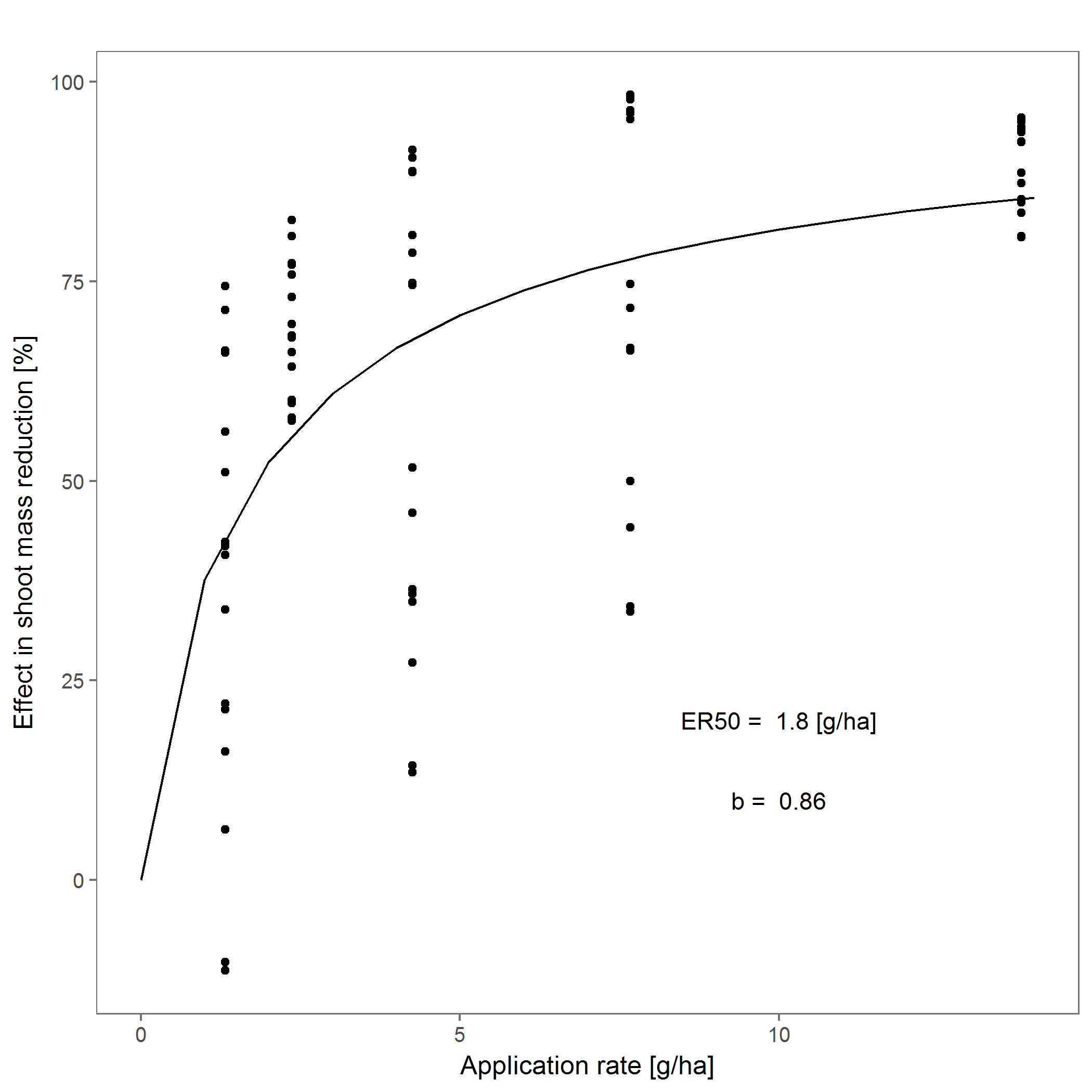
**

Figure D.9: Effects on the fresh weight (% reduction of fresh weight) of *L. hispidus* in the monoculture treatment 4 weeks after application, when sprayed with different application rates of the selective herbicide Monitor®. Points show the empirically measured data and the line the estimated dose response function, with the predictors for the ER50 value and the slope b.


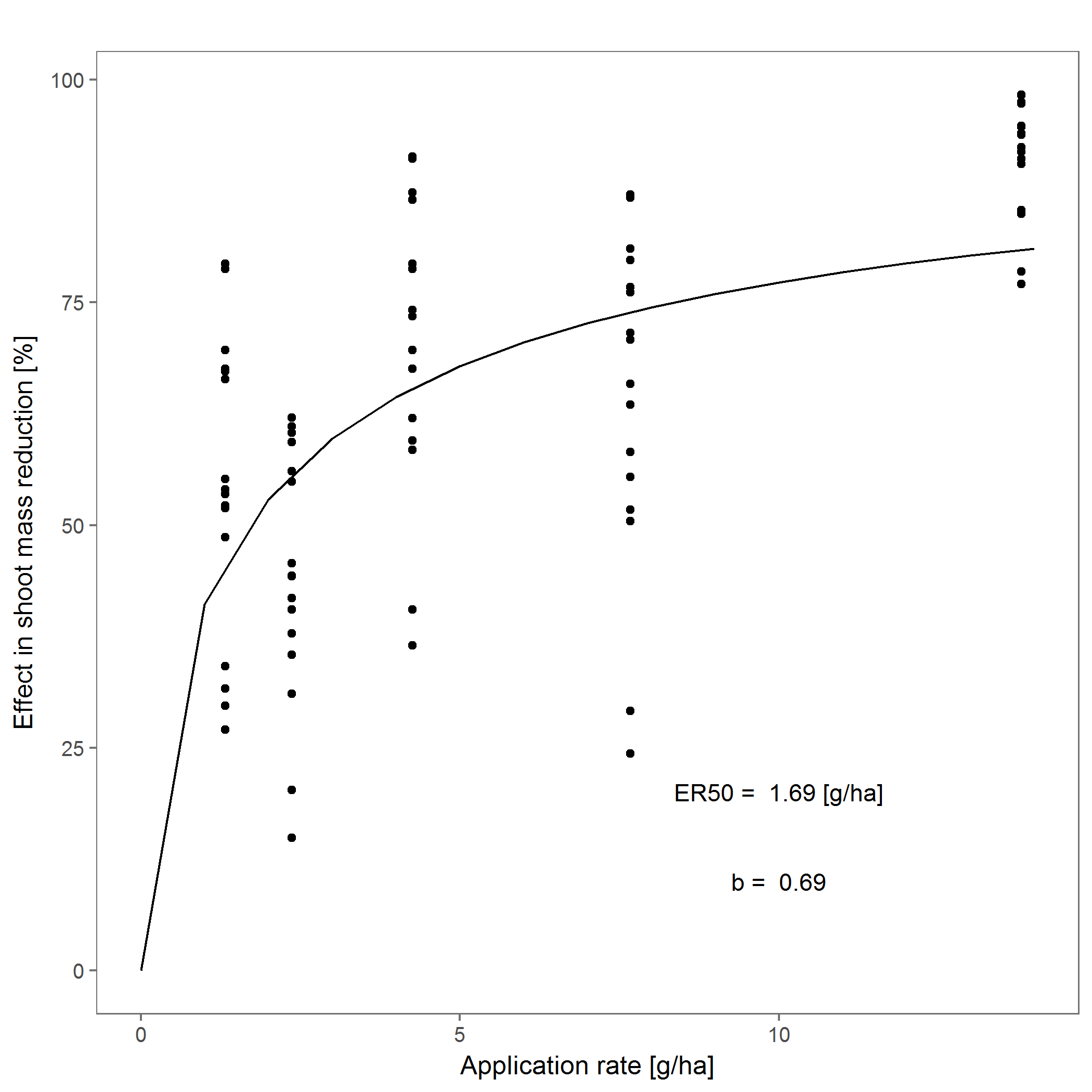


Figure D.10: Effects on the fresh weight (% reduction of fresh weight) of *S. nutans* in the monoculture treatment 4 weeks after application, when sprayed with different application rates of the selective herbicide Monitor®. Points show the empirically measured data and the line the estimated dose response function, with the predictors for the ER50 value and the slope b.


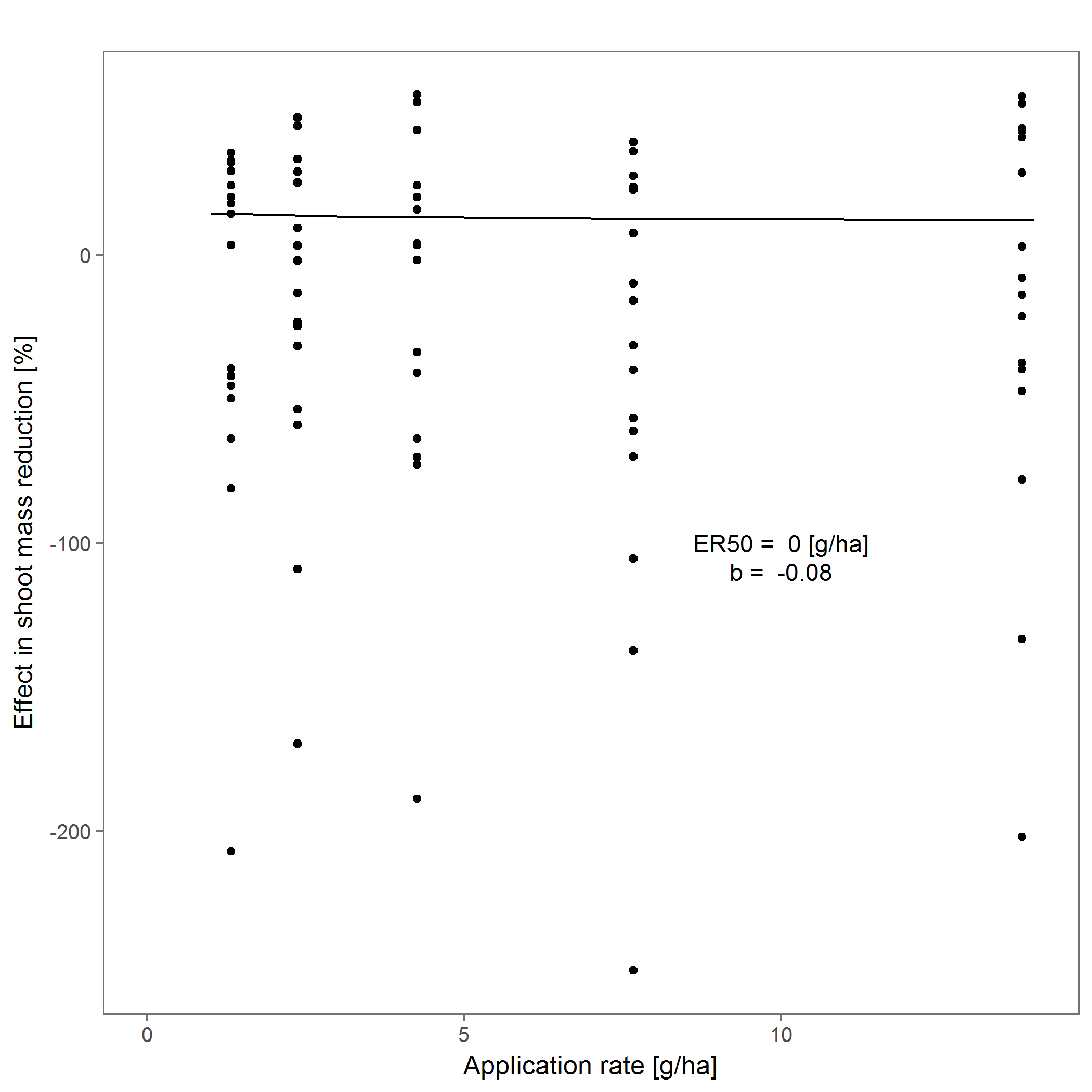


Figure D.11: Effects on the fresh weight (% reduction of fresh weight) of *T. pratense* in the monoculture treatment 4 weeks after application, when sprayed with different application rates of the selective herbicide Monitor®. Points show the empirically measured data and the line the estimated dose response function, with the predictors for the ER50 value and the slope b.

## Appendix E: Results for all application rates


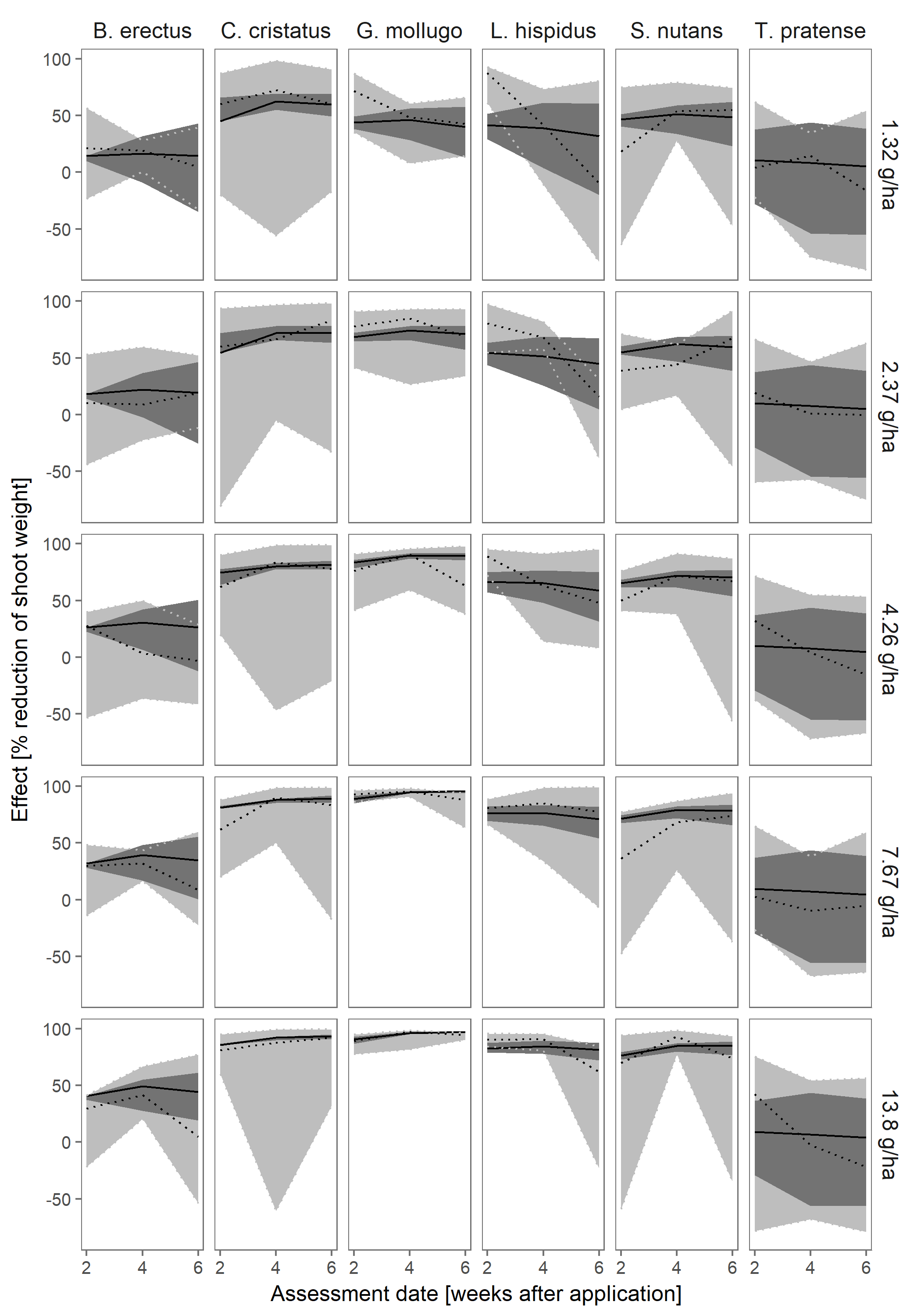


Figure E.1: Effects on species specific shoot masses (treatment/control) in the monocultures after application of the selective herbicide Monitor®. Black solid lines represent the median of the model predictions and dark grey ribbons show the upper and lower 2.5th percentile of the predictions. Dotted black lines show the empirically measured median and grey ribbons and dotted grey lines the upper and lower 2.5th percentile of these.


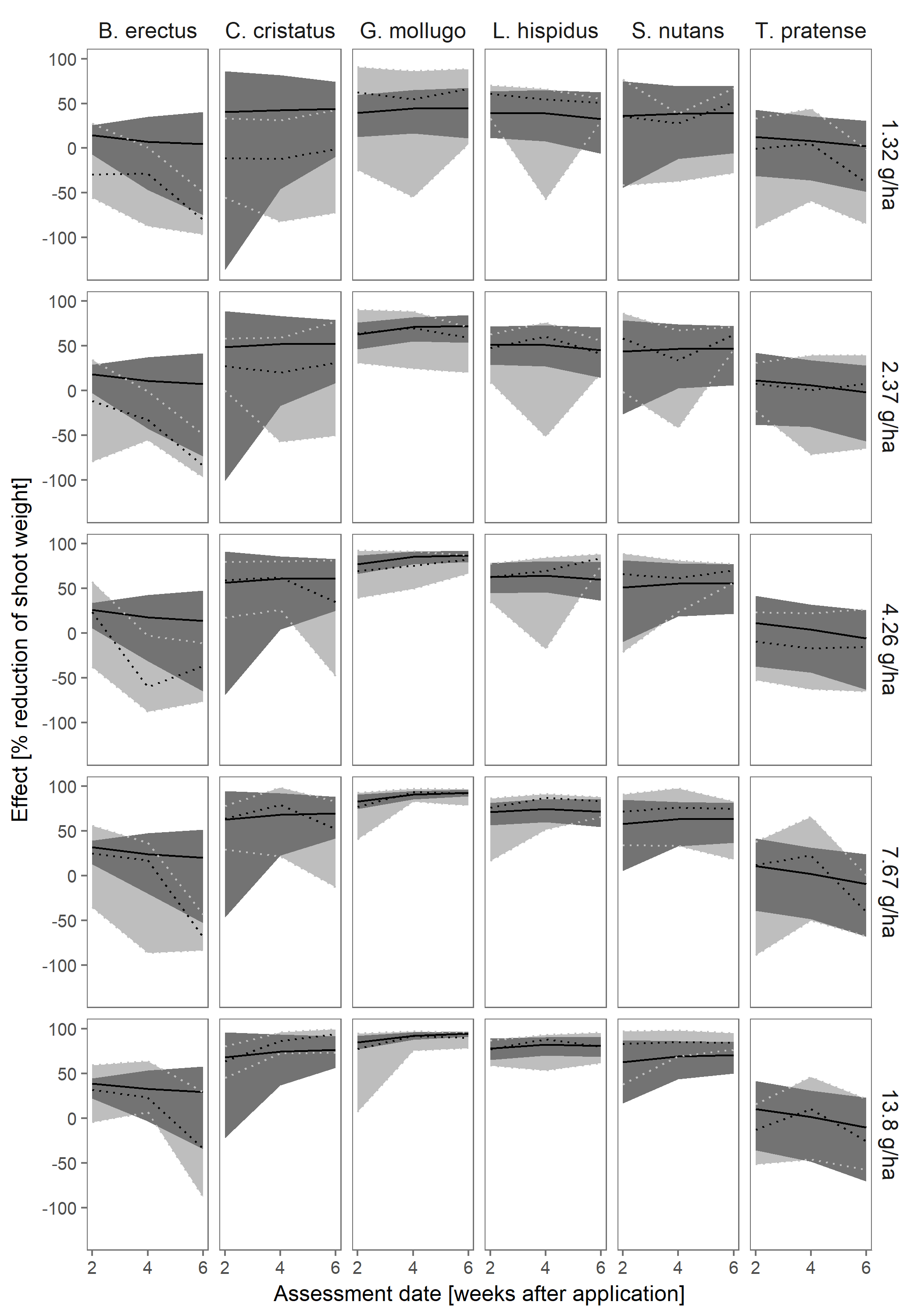


Figure E.2: Effects on species specific shoot masses (treatment/control) in the artificial communities after application of the selective herbicide Monitor®. Black solid lines represent the median of the model predictions and dark grey ribbons show the upper and lower 2.5th percentile of the predictions. Dotted black lines show the empirically measured median and grey ribbons and dotted grey lines the upper and lower 2.5th percentile of these.


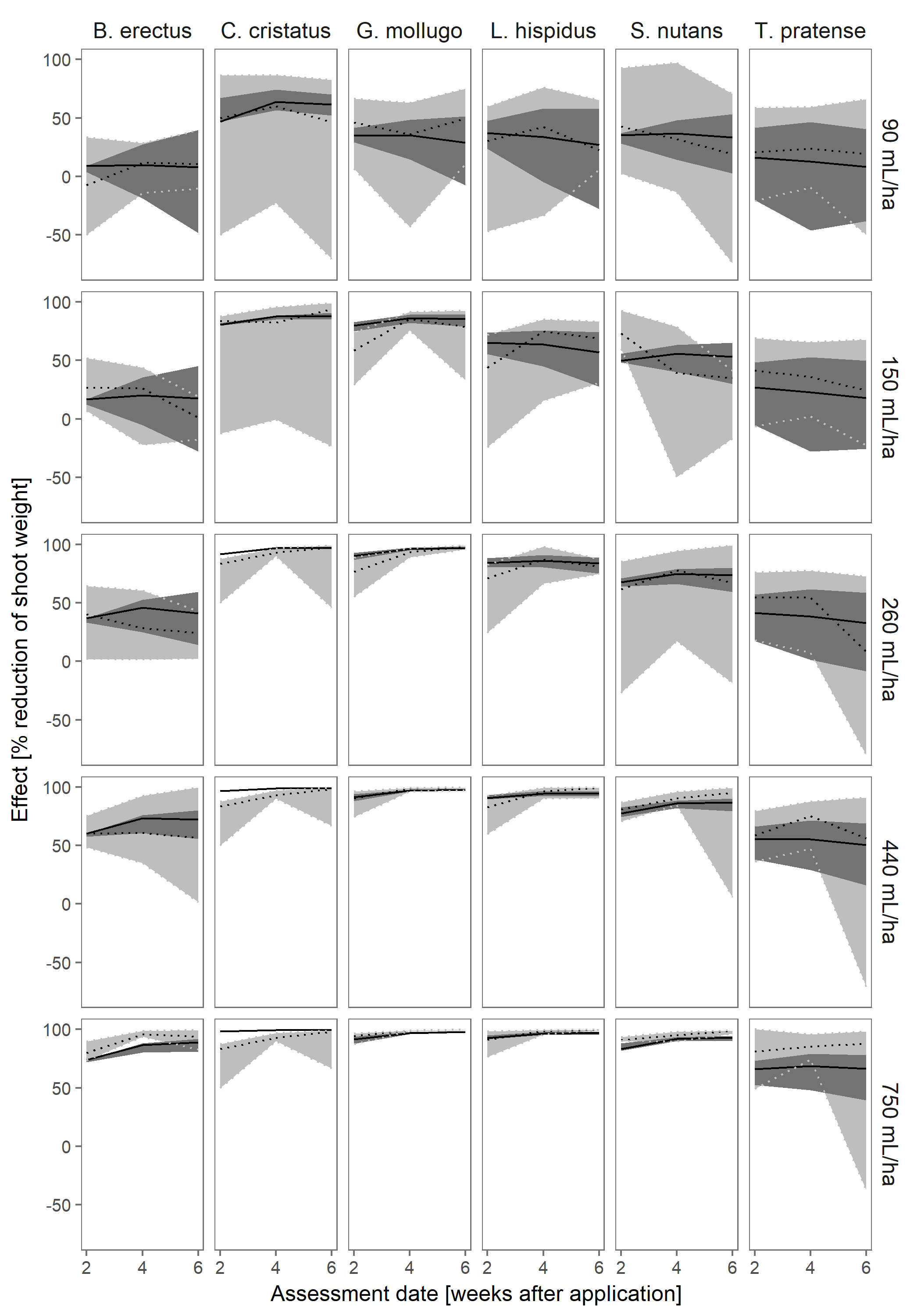


Figure E.3: Effects on species specific shoot masses (treatment/control) in the monocultures after application of the broad spectrum herbicide RoundUp®. Black solid lines represent the median of the model predictions and dark grey ribbons show the upper and lower 2.5th percentile of the predictions. Dotted black lines show the empirically measured median and grey ribbons and dotted grey lines the upper and lower 2.5th percentile of these.


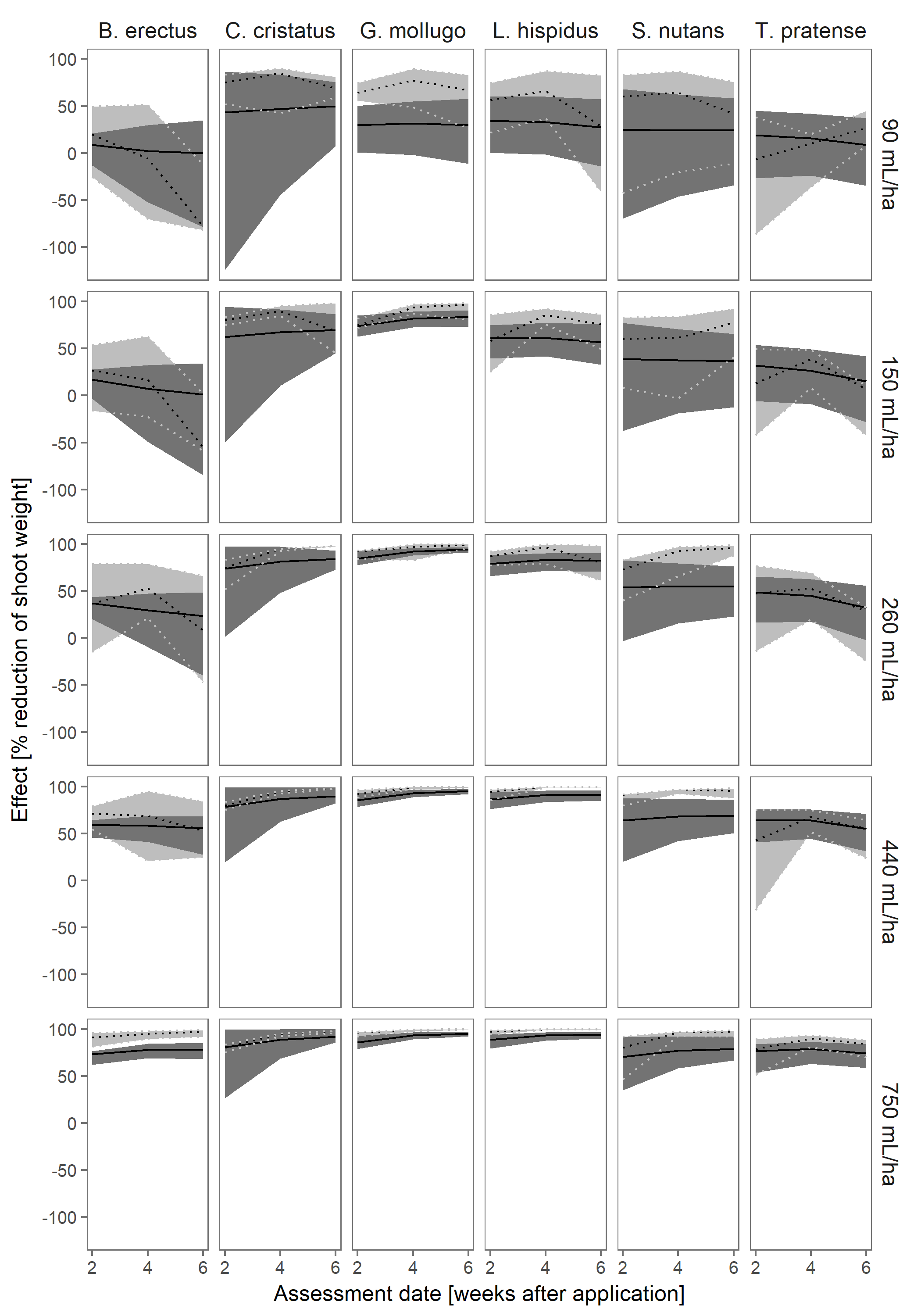


Figure E.4: Effects on species specific shoot masses (treatment/control) in the artificial communities after application of the broad spectrum herbicide RoundUp®. Black solid lines represent the median of the model predictions and dark grey ribbons show the upper and lower 2.5th percentile of the predictions. Dotted black lines show the empirically measured median and grey ribbons and dotted grey lines the upper and lower 2.5th percentile of these.

## Appendix F: Comparison of plant growth modeled in single pots, monocultures and in the empirical monoculture


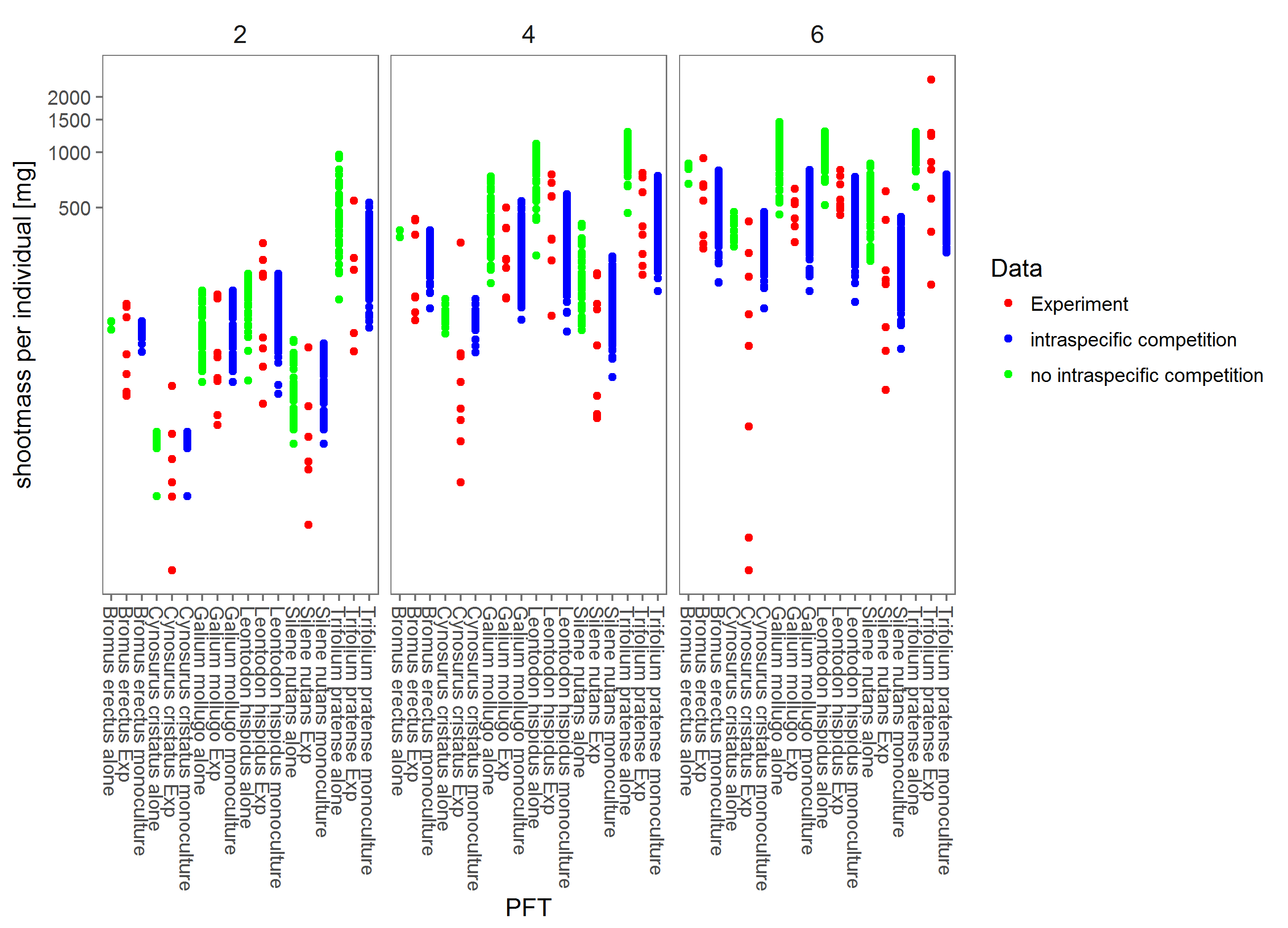


Figure F.1: Simulated shoot masses for plant individuals grown in single pots (no intraspecific competition), in monocultures (intraspecific competition) vs. shoot masses measured in monoculture controls (Experiment).
